# Female Oncofertility: Current Understandings, Therapeutic Approaches, Controversies, and Future Perspectives

**DOI:** 10.3390/jcm10235690

**Published:** 2021-12-03

**Authors:** Kim Cat Tuyen Vo, Kazuhiro Kawamura

**Affiliations:** 1 Graduate School of Medicine, International University of Health and Welfare, Narita, Chiba 286-8686, Japan; votuyen9788@gmail.com; 2 Department of Obstetrics and Gynecology, School of Medicine, International University of Health and Welfare, Narita, Chiba 286-8686, Japan

**Keywords:** chemotherapy, fertility preservation, gonadotoxic, oncofertility, oocyte quality, ovarian reserve, premature ovarian insufficiency, radiotherapy

## Abstract

Recent advances in early detection and oncological therapies have ameliorated the survival rate of young cancer patients. Yet, ovarian impairment induced by chemotherapy and radiotherapy is still a challenging issue. This review, based on clinical and lab-based studies, summarizes the evidence of gonadotoxicity of chemoradiotherapy, the recent approaches, ongoing controversies, and future perspectives of fertility preservation (FP) in female patients who have experienced chemo- or radio-therapy. Existing data indicate that chemotherapeutic agents induce DNA alterations and massive follicle activation via the phosphoinositide 3-kinase (PI3K)/Akt signaling pathway. Meanwhile, the radiation causes ionizing damage, leading to germ cell loss. In addition to the well-established methods, numerous therapeutic approaches have been suggested, including minimizing the follicle loss in cryopreserved ovarian grafts after transplantation, in vitro activation or in vitro growing of follicles, artificial ovarian development, or fertoprotective adjuvant to prevent ovarian damage from chemotherapy. Some reports have revealed positive outcomes from these therapies, whereas others have demonstrated conflictions. Future perspectives are improving the live birth rate of FP, especially in patients with adverse ovarian reserve, eliminating the risk of malignancy reintroducing, and increasing society’s awareness of FP importance.

## 1. Introduction

In the past two decades, substantial advances in early diagnosis and cancer treatment have resulted in an approximately 80% 5-year survival rate in young oncological patients [1,2], leading to a rise in number of female childhood cancer survivors (CCS) [3]. However, oncologic treatment usually requires extensive chemotherapy and/or radiotherapy, which are indicated to be distinctively ovotoxic, resulting in premature ovarian insufficiency (POI) and consequent infertility [4,5,6,7]. Approximately, 30% of children who were exposed to chemo- and/or radio-therapy develop gonadal dysfunction [8]. The incidence of POI in CCS is estimated as high as 8–10% [4].

Although the mechanism is not fully elucidated yet, current data demonstrate that chemotherapeutic agents, especially alkylating ones, interfere with DNA replication and cell division [9], massively activate the primordial follicles (PFs) [10,11], cause stroma atresia [12], and damage the vascularity in ovaries [13]. The radiation is also harmful to oocytes as its low dose of less than 2 Gy can destroy 50% of primordial follicles [7,14].

This fertility-compromised status has been well-documented to cause emotional distress and poor quality of life [15,16,17,18]. It was reported that there will be approximately 100 million women at risk of chemotherapy-induced ovarian impairment in 2025 [19]. In this context, preserving the fertility and quality of life of CCS has received considerable concerns. During the last two decades, FP with several effective approaches has been significantly developed, and represents a beneficial option to help hundreds of oncological women have genetic offspring. Furthermore, increasing studies are attempting to clarify the mechanisms and outcomes of chemo- and radio-therapy impacts on the ovarian reserve and oocyte quality, to develop protective methods as well as to improve therapeutic approaches in FP.

The purpose of this review is to summarize the published evidence describing the deleterious effects of chemo- and radio-therapy and the related mechanisms. Furthermore, the current options of FP, the preventive approaches to protect the fertility for female CCS as well as the existing debates are also included.

## 2. Impact of Chemo- and Radio-Therapy on Follicle Quantity

### 2.1. Clinical Data Describing the Impact of Chemo- and Radio-Therapy on Ovarian Function

A growing number of studies have demonstrated that the pregnancy rate and live birth rate in female CCS are lower compared to those of their siblings and general population controls. The results of these studies are summarized in Table 1.

In a longitudinal study including 66 patients undergoing chemotherapy, the AMH levels were decreased significantly (0.90 ± 1.55 compared to 2.61 ± 2.20 ng/mL before treatment) after chemotherapy using BEACOPP protocol (Bleomycin, Etoposide, Doxorubicin, Cyclophosphamide, Vincristine, Procarbazine, and Prednisolone) during a following period of 16.8 ± 9.3 months. In the ABVD protocol (Doxorubicin, Bleomycin, Vinblastine, and Dacarbazine), the AMH levels prior and after treatment were not statistically different (4.38 ± 3.39 vs. 4.27 ± 3.09 ng/mL, *p* = 0.753) [20]. Another study recorded the rates of diminished ovarian reserve and POI after chemotherapy as 39% (35/90) and 21% (19/90), respectively [21].

According to a population-based analysis, the overall likelihood of pregnancy in female CCSs aged under 40 is a 38% lower than that in the general population of women [4]. In another study, laboratory results show impairment in the concentration of female gonadal-related hormones (LH, FSH, and estradiol) in 24.3% (97/444) of female CCSs who were younger than 40 years of age [23]. According to a cohort study of 552 female CCSs in Sweden, the hazard ratio (HR) for having a first live birth in CCSs with malignancy of the eye, central nervous system tumors, and leukemia, is statistically lower than in healthy controls [29]. In 2930 CCSs, 110 survivors encountered POI with the value of 10.3 as an odds ratio compared to their healthy siblings, resulting in lower birth rates in their thirties [28]. In another report, the relative likelihood of 5149 CCSs achieving pregnancy is 0.81 (95% CI, 0.73 to 0.90; *p* < 0.001) compared with that of female siblings [38]. In a large sample cohort study including 5298 female five-year cancer survivors, their likelihood of having a pregnancy is significantly lower than their siblings (HR 0.85, 95%: 0.74–0.98; *p* = 0.023) [5]. The effects of the alkylating drugs and cisplatin on ovarian functions show a dose-dependent manner [5]. In a systematic review including 5607 female CCSs, the prevalence of amenorrhea ranges from 0% to 83% [6]. Exposure to alkylating agents and older age at treatment are detected as the decisive factors contributing to ovarian dysfunction [6].

### 2.2. Mechanism of Chemo- and Radio-Therapy Induction of Follicular Loss

To develop new therapies of FP and fertoprotective agents, numerous studies have described the possible mechanisms in which chemo- and radio-therapy induce ovarian damage. As typical chemotherapy protocols often consist of several agents, determining the ovarian impairment caused by each type of antitumor drug in clinical studies is challenging. Consequently, the conceptual effect of a single drug on the ovary is usually clarified by in vitro cell culture, ovarian tissue culture, or in vivo animal models, and human ovarian tissue culture followed by xenotransplantation [39,40].

**Figure 1:**
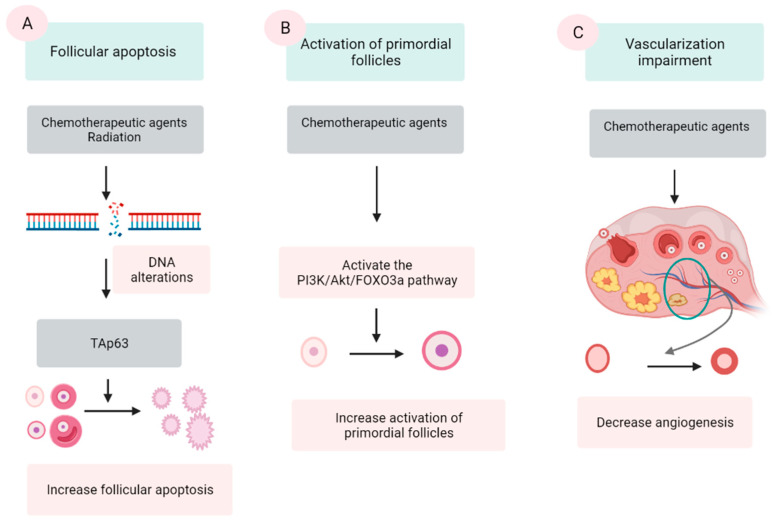
Three mechanisms of chemo- and radio-therapy-induced follicular quantity depletion: enhancement of apoptosis, accelerated activation of PFs. (**A**) DNA alterations induced by chemotherapeutic agents and radiation activates TAp53 protein, leading to the apoptosis. (**B**) Chemotherapeutic agents activate the phosphoinositide 3-kinase (PI3K)/Akt/forkhead box protein O3a (FOXO3a), which in turn induce the activation of PFs, resulting in the extensive loss of PFs. (**C**) Chemotherapeutic agents impair the epithelial tissue of vessels in the ovary, resulting in a reduction in the vascularization.

Chemotherapeutic agents are generally divided into five categories: alkylating agents (cyclophosphamide, procarbazine, and busulfan), platinum-based compounds (cisplatin and carboplatin), anthracycline antibiotics (doxorubicin and bleomycin), antimetabolites (methotrexate and 5-fluorouracil), and vinca alkaloids (vincristine and vinblastine). The first three groups are demonstrated to damage ovaries by inducing DNA alterations, leading to follicular apoptosis [41,42]. Among these, alkylating agents are supposed to be most ovotoxic, causing significant follicular loss [6,43]. The last two groups are indicated to have a low risk to ovarian function [42]. However, some data show that vinca alkaloids, due to their suppression of microtubule dynamics, induce a vascular impairment, leading to ovarian dysfunction [44,45]. Three major mechanisms were proposed by several scientific groups (Figure 1).

#### 2.2.1. Follicular Apoptosis after Chemotherapy

The extensive apoptosis of ovarian follicles, especially PFs, after DNA alterations and/or oxidative stress is the most commonly described mechanism in chemotherapy-induced ovarian failure [41,46,47]. Several agents in the antitumor protocols, especially alkylating agents, are demonstrated to cause DNA lesions in both oocyte and granulosa cells (GCs). Among these lesions, double-stranded DNA breaks are among the most severe [39,42]. The accumulation of DNA strand breaks that could not be repaired by the DNA repairing system induces the apoptotic intracellular pathways, resulting in cellular apoptosis [42]. p63 protein (TAp63 isoform), Bcl2-associated X (BAX) protein and the BCL-2 antagonist killer (BAK) protein activator, is the major protein that mediate this mechanism [48,49].

Culture with cyclophosphamide [46] as well as in vivo cyclophosphamide injection [50] of mice’s ovaries induces DNA damage and subsequent follicle apoptosis. Cisplatin also causes DNA impairment and PFs’ apoptosis in both newborn and adult mouse ovaries [41]. Cyclophosphamide treatment substantially decreases the number of PFs, primary follicles, and secondary follicles with an elevated number of atretic follicles compared with control animals [13]. In another experiment, intraperitoneal injection of cyclophosphamide and cisplatin caused a significantly destructive effect on the PFs pool [51]. However, in mice with gene deletion of PUMA, a member of BCL-2 protein family, the PFs are retained after the treatment by both cyclophosphamide and cisplatin [51]. In a human ovarian xenograft model, cyclophosphamide [52], cisplatin [53], and doxorubicin [39] elevated DNA double-stranded breaks and resulted in a significant follicle loss.

The effect of antitumoral drugs on ovarian function is the follicle-specific magnitude and is associated with the category of the drugs [12]. Some studies have declared that apoptosis occurred only in GCs of growing follicles, but not in PFs by TUNEL staining after treatment of cyclophosphamide or cisplatin [54,55]. Other results insist that TUNEL and/or γH2AX staining are positive in oocytes but not in the GCs of PFs [46,50,56]. In another experiment, culturing ovaries with cisplatin or carboplatin decreases the number of follicles of all stages, but the most obvious reduction is observed in PFs [57]. One study reported that culturing of neonatal mice ovaries in cisplatin or doxorubicin significantly decreased the number of follicles at all stages [58]. However, the apoptosis evidence in the TUNEL analysis is not positive in the PFs, only in the growing follicles [58].

#### 2.2.2. Activation of PFs Induced by Chemotherapy

An additional suggested mechanism for ovarian impairment after oncological treatment is the accelerated activation of PFs. Several scientific groups have confirmed that chemotherapy causes massive activation of PFs in affected ovaries via a phosphoinositide 3-kinase/protein kinase B/forkhead box protein O3a (PI3K/AKT/FOXO3a) pathway [54,59,60,61].

Mice administered intraperitoneally with cisplatin show a substantially decreased number of PFs along with higher numbers of early growing follicles and the signal of the key proteins in the PTEN/Akt/FOXO3a [59]. Other studies also revealed increased phosphorylation of Akt, mTORC, and downstream proteins followed by PF reduction in cyclophosphamide-treated mice [54,62]. In mice, doxorubicin causes detrimental effects on ovaries through both atresia and overactivation in PFs [63]. The same effects are found in mice treated with cisplatin [59]. In another experiment using neonatal mouse ovaries cultured with cisplatin or doxorubicin, PFs decrease without the evidence of apoptosis in TUNEL analysis, suggesting the etiology of PF reduction by overactivation [58]. In terms of human ovarian follicles, exposure to cyclophosphamide metabolites in vitro also induces PFs’ activation [61]. Furthermore, a cohort study of 96 female CCSs who were treated with alkylating agent revealed PFs activation in vivo and a remarkably suppressed nuclear expression of FOXO3a occurring in ovaries of these patients [64].

In consistence with this hypothesis, many experiments have indicated that inhibiting the PI3K pathway by several agents including rapamycin, ammonium trichloro (dioxoethylene-o,o′) tellurate (AS101), anti-Müllerian hormone (AMH), and melatonin, could prevent PF’s activation after chemotherapy [54,55,60,65,66,67,68].

Although this mechanism has been widely accepted, recent literature has raised the argument that activating PFs might not be the major or a specific cause of chemotherapy-induced PF loss [11]. Accordingly, the authors doubt that a growing follicles to PFs ratio calculation were not the correct parameter for a sign of PFs’ activation, because elimination of PFs could occur due to a deleterious effect. In an experiment, after culture of intact mouse ovaries with the metabolite agent cyclophosphamide, the number of PFs decreased along with increased levels of apoptotic markers BAX and cPARP. Meanwhile, there was no significant change in the number of primary follicles. In combination with the TUNEL staining’s results, this study indicated that the decrease in PFs was not due to their activation but the apoptosis in PFs [46]. A recent study demonstrated the depletion of PFs after cyclophosphamide exposure in a human ovarian xenograft model, utilizing triggering of proapoptotic pathways without evidence of PFs activation, and indicated that apoptosis was the main mechanism of PFs’ depletion [69].

#### 2.2.3. Vascularization Impairment

Another proposed mechanism is the alteration in angiogenesis and stroma supporting the gonadal cells after exposure to chemotherapeutic agents [70,71]. Cyclophosphamide treatment shows induction of inflammation and enhanced expression of stromal cell-derived factor 1 (SDF-1), a factor related to follicular atresia, which presents in the granulosa, theca cells, and luteinized cells [72]. In human ovaries, histological analyses of ovaries from cancer survivors show the presence of damaged cortical blood vessels and proliferation of small vessels (neovascularization). Furthermore, the muscular layer in blood vessels becomes thicker, leading to limited blood circulation. The cortex presents fibrotic focal areas along with disappearance of follicles [73]. During in vivo monitoring, an evident reduction in ovarian circulation and spasm of small vessels are noted after the administration of doxorubicin [74]. In vitro human ovarian tissue culture with doxorubicin followed by xenograft to immunodeficient mice has a lower vascular density and higher microvascular compromise compared with controls [39]. In addition, one study assessing human ovarian tissue shows that both alkylating and nonalkylating drugs affect ovarian stromal function, leading to a substantial decrease in estradiol production [75].

#### 2.2.4. Radiation

Regarding radiotherapy, the human oocyte is very sensitive to radiation, and a dose as low and less than 2 Gy for pelvic radiation can destroy 50% of PFs [76]. The position of radiation is one determinant factor of the degree of ovarian damage. The rate of POI in patients who experienced total body radiation and pelvic irradiation are 90% and 97%, respectively [77]. In addition, factors such as patient age and radiation dose are also important contributors [73,77]. Aging patients are more vulnerable to radiation compared with younger girls, due to the age-related decline in the follicle population [78]. The dose causing ovarian dysfunction in children is 1–2 Gy, whereas in adults it is as low as 0.4–0.6 Gy [79]. Based on an analysis from five centers conducting ovarian tissue cryopreservation (OTC), the live birth rates after OTC in patients undergoing pelvic irradiation reduced significantly in a dose-dependent manner [80].

The proposed mechanism of follicle depletion is the radiation-provoked ionizing damage of DNA [81]. This alteration also activates TAp63 protein, leading to destruction of PFs [49]. In terms of late effects, vasculature damage and stromal fibrosis following tissue hypoxia could be another mechanism [77]. This can result in ovarian atrophy and subsequent tissue dysfunction [73].

## 3. Impact of Chemo- and Radio-Therapy on Follicle Quality

### 3.1. Studies Describing Impact of Chemo- and Radio-Therapy on Follicle Quality

In addition to the deleterious effects on ovarian reserve, chemo- and radio-therapy also decrease oocyte quality. In human ovaries, a significant increase in abnormalities in GCs’ nuclei (*p* < 0.05–0.0001) and oocyte vacuolization (*p* < 0.0001) is noted by fluorescence microscopy imaging after exposure to chemotherapeutic agents, especially alkylating agents [82]. In addition, anthracycline compounds are reported to induce oxidative stress and mutations in oocytes [42,83].

Similarly, postchemotherapy patients’ ovaries have a higher rate of oocyte vacuolization and detachment of the oocyte from GCs when compared with prechemotherapy ones (34.3 ± 5.3% vs. 26.1 ± 4.2%) [84]. The malignancy itself has negative impacts on the oocyte quality. In detail, an oncological patient group had a notably elevated number of abnormal oocytes in comparison to a control group [85].

### 3.2. The Mechanism of Chemo- and Radio-Therapy on Follicle Quality

If DNA alterations are not repaired efficiently, mutagenic oocytes can be formed [10]. Cisplatin treatment causes significant morphological abnormalities in oocytes of primary follicles at all doses [58]. A significant increase in the percentage of morphological abnormalities in GCs in both transitional and primary follicles is also reported after doxorubicin exposure [58]. For both drugs, the highest doses can damage follicles in both the oocyte and GCs [58]. In another experiment, cisplatin increased the rate of aneuploidy in oocytes, leading to early embryonic death [67].

Regarding radiation, exposure to doses below effective sterilizing dose still causes DNA damage, leading to genetic disorders in oocytes [49].

## 4. The Therapeutic Options for FP

### 4.1. Oocyte and Embryo Cryopreservation

Since the early stage of FP, oocyte and embryo cryopreservation have been well-established and used worldwide as FP methods. The American Society for Reproductive Medicine (ASRM) removed the experimental label for oocyte cryopreservation in 2012 [86]. According to the European Society of Human Reproduction and Embryology (ESHRE) guidelines, oocyte/embryo cryopreservation should be offered as an established option for FP [87]. Oocyte cryopreservation consists of ovarian stimulation, mature oocyte retrieval, and cryopreservation. Embryo cryopreservation requires in vitro fertilization (IVF) with husband sperm as a further step before cryopreservation.

For ovarian stimulation, the GnRH antagonist protocol is recommended for its feasibility in urgent situations [87]. It is more beneficial than the GnRH agonist protocol because of the short duration of stimulation, and the comparable retrieved oocyte number and pregnancy rate [88,89,90]. According to a national analysis in young breast cancer patients, ovulatory trigger using GnRH agonist yields superior outcomes including higher numbers of retrieved mature oocytes and cryopreserved embryos in comparison with the hCG trigger [91]. Furthermore, a systematic analysis demonstrates that GnRH agonist trigger reduces the risk of ovarian hyperstimulation [92]. Regarding cryopreservation, the recent implementation of vitrification has yielded positive outcomes in oocyte/embryo cryopreservation. A range of evidence has suggested that the oocyte/embryo vitrification and thawing method yielded higher pregnancy and live birth rates than slow freezing [93,94,95]. Other improvements in ovarian stimulation have also contributed to the success of oocyte/embryo cryopreservation. For instance, to minimize the duration of stimulation and increase the number of retrieved oocytes, random-start cycles and Doustim protocol (repetition of two ovarian stimulations within the same menstrual cycle protocol) have emerged, and have demonstrated to be more beneficial. There is no clinically important difference in these procedures regarding the number of retrieved mature oocytes, total oocytes retrieved, fertilization rate, or the number of cryopreservation embryos when compared to the conventional ovarian stimulation protocols [96]. In addition, the combination of letrozole during ovarian stimulation with gonadotropins decreases substantially the peak estradiol levels without accompanying negative effects on the oocyte maturation, to offer the better condition in cases of estrogen-sensitive cancers (e.g., breast and uterine endometrial cancers) [97]. Another study also confirms the long-term safety of this combination in breast cancer patients after a period of five-year follow-up [98]. During the last decade, the implementation of in vitro maturation (IVM) has also increased the chance of successful pregnancy. Immature oocytes can be retrieved simultaneously with the mature oocyte, and subsequently cultured in vitro for 24–48 h to mature into metaphase II oocytes, maximizing the number of obtained fertilizable oocytes [99,100]. In another aspect, oocyte and embryo cryopreservation provides a chance for preimplantation genetic testing during IVF procedure, which helps to eliminate the possibility of malignancy transmission to their offspring in genetic-related cancer patients [101,102,103]. Otherwise, the IVF procedure using a donor oocyte and subsequent embryo cryopreservation is an alternative option, which prevents occurrence of genetically based tumors in these patients’ offspring [104]. The live birth rate with oocyte/embryo cryopreservation depends on the age of patients and the number of cryopreserved oocytes/embryos [81]. The live birth rate is reported after oocyte cryopreservation to range from 32.6% [105] to 42.1% [106]. Per oocyte, the live birth rates were 8.7% (women < 30 years) and 1.1% (women 43–44 years) [107]. Recent data indicate that having 10–12 oocytes leads to reasonable cumulative live birth rates up to 61.9% and 43.4% in patients <35 years of age and ones >35 years of age, respectively [106,108]. In breast cancer patients, the controlled ovarian stimulation and oocyte cryopreservation before antitumor therapeutics granted 13 successful live births among 332 patients, without increasing the cancer recurrence rate or mortality rate [109]. In a study including 1073 women (1172 vitrification cycles) diagnosed with cancer undergoing oocyte cryopreservation, after a mean storage time of 4.1 ± 0.9 years, the oocyte survival rate is 81.8%, and after transferring a mean number of 1.4 ± 0.1 embryos, the clinical pregnancy rate and live birth rate are 41.4% and 31.2%, respectively [106]. In terms of embryo cryopreservation, the live birth rate per one transferred embryo in breast cancer patients is comparable to one in noncancer population (45.0% vs. 38.2%) [110]. Similar to general infertile patients, the pregnancy rate from embryo cryopreservation in oncological patients decreases with increasing age when they cryopreserve embryos [111,112].

Oocyte/embryo cryopreservation might not be an option for patients who need urgent oncological treatment, or pediatric patients because of their premenarcheal status [113]. In these cases, OTC and other options could be suggested.

### 4.2. OTC

OTC comprises the removal of ovarian tissue and cryopreservation of cortical fragments, which are later orthotopically transplanted to restore both endocrine and fertility function of ovaries [114,115]. The major advantages of OTC are the short timeframe required for its performance and the possibility to preserve both fertility and endocrine function. OTC is currently a unique option for prepubescent girls and patients whose gonadotoxic treatment delay or conventional ovarian stimulation is contraindicated [116,117]. Moreover, because of the rich source of PFs in young patients, OTC could yield the greatest fertility potential in CCS [115,118,119,120]. OTC could also be a beneficial option for patients who underwent chemotherapy because chemotherapy is no longer a contraindication to freezing, as demonstrated by previous studies [80].

Since the first of such pregnancies was reported in 2004 [121], more than 200 live babies have been born [122]. Overall, the rate of restoration of ovarian endocrine function is more than 90% in cases after transplantation within 4–9 months [122,123]. In a large sample size report including 111 patients in five major centers, the pregnancy and live birth rates were 29% and 23%, respectively [124]. Another big sample study in a single center reported on conducting OTC in 418 prepubertal children over 20 years. However, no pregnancy was reported in this study [125]. The first birth obtained after OTC and transplantation of ovarian tissues to a prepubertal patient was reported in 2015 [126]. In a large series report from five leading European centers, the live birth rates were 30% and 21%, respectively, among those conceiving naturally and those undergoing in vitro fertilization (IVF) with a low rate of malignant relapse [80]. In general, the live birth rate is reported to be about 40% among survivors who are <36 years of age [107]. Consequently, the updated guidelines of the ASRM and ESHRE recommend that OTC should be considered as an established procedure to be offered to selected patients for FP purposes [127,128]. Moreover, recent data including results from five European centers points that chances of pregnancy success are not impaired, even if OTC is conducted after chemotherapy [80]. In addition to the positive reproductive outcomes, OTC also helps to recover the ovarian endocrine functions manifested by the restoration of menstrual cycles and improved hormonal profile [129]. The recovery rate is reported to be 70% in survivors after transplantation of ovarian tissue [129]. Interestingly, functional transplanted ovarian tissue is sustained during a long period of 8 years in two CCS cases [130].

OTC is currently conducted by conventional slow freezing or vitrification [131,132]. A systematic review and meta-analysis suggest the superiority of vitrification to slow freezing regarding clinical outcomes in survival rates for oocytes, cleavage-stage embryos, and blastocysts [93]. However, slow freezing is shown to be more beneficial than vitrification in OTC. Cryopreservation of human ovarian tissue by slow freezing was reported to produce tissues with more remaining PFs compared with vitrification [133]. Looking at a number of studies, slow freezing is recognized to be superior in preservation of follicle quality in OTC [134,135,136]. According to the EHSRE guideline, the slow-freezing protocol should be used for OTC as it is well-established and considered as standard [87].

In recent years, along with the development of the in vitro maturation (IVM) approach in infertility treatment, several scientific groups have tried to combine IVM to the OTC, since early antral follicles could not survive after cryopreservation. Accordingly, immature oocytes which are obtained transvaginally or retrieved from ovarian tissue “ex vivo” could mature in vitro to obtain mature oocytes ready for IVF, increasing the possibility of success [137,138]. In recent data, a combination of OTC and an “ex vivo” IVM of retrieved oocyte results in an outcome comparable to that of oocytes obtained after ovarian stimulation before cancer treatment, regarding the number of mature MII oocytes and live births [138]. The recent introduction of the biphasic with a prematuration step by using c-type natriuretic peptide (CNP) improves markedly the oocyte competence and coordination between the oocyte and GCs [139]. CNP has been demonstrated to be able to temporarily maintain meiotic arrest in oocytes by activating the natriuretic peptide receptor B in cumulus cells [140,141]. Culturing CNP during the pre-IVM step improves mitochondrial function and developmental competence of oocytes, resulting in IVM success in several animal species [142,143,144,145,146] as well as in humans [147]. Furthermore, the addition of supplements during the pre-IVM period enhances the oocytes’ maturation during the subsequent IVM culture step [148]. For instance, the treatment of cyclic adenosine 3′5′-monophosphate (cAMP) modulators prior to IVM improves murine oocyte maturation [149]. Similarly, the utilization of 3-isobutyl-1-methylxanthine in the pre-IVM step results in a higher rate of meiotic progression of the germinal vesicle stage, nuclear maturation, and subsequent embryonic development in bovines [150]. Furthermore, the treatment of dibutyryl-cAMP with L-ascorbic acid improves the developmental competence of porcine oocytes [151].

However, there are some inquiries that need to be improved in OTC. Firstly, there is significant follicle loss due to ischemia after transplantation of ovarian tissue, reducing the lifespan of the graft [152,153]. It is reported that around 80% of ovarian follicles are lost during the OTC-followed-by-transplantation procedure [154]. To enhance the neoangiogenesis after grafting, several agents are suggested for use during transplantation, including angiogenic and antiapoptotic factors, antioxidants, and adipose-derived stem cells [155,156,157,158]. A recently published study revealed that simvastatin and fibrin clots promoted vascularization of the human ovarian tissue after grafting [159]. Secondly, the risk of reintroducing malignant cells after autotransplantation to a cancer survivor remains a concerning issue [160]. Some groups have conducted ovarian tissue transplantation in leukemia patients and no relapse has been reported [80,122,161]. However, in the recent review, 9 out of 230 CCS who underwent OTC experience the recurrence of malignant diseases, although a relationship with the transplantation procedure was not found [122]. At the moment, OTC is not recommended in hematologic malignancy, ovarian cancer, or cancers that metastasize to the ovary [116,162]

### 4.3. In Vitro Activation (IVA) of PFs

Due to the low follicle reserve in advanced age patients [163], OTC is not recommended to women with advanced age or low ovarian reserve because of short lifespan of the graft. In these cases, a combination of in vitro follicle activation (IVA) of PFs and OTC was developed to maximize the chance of pregnancy achievement. In this procedure, obtained ovarian tissue is fragmented into cubes at the size of 1 × 1 × 1 mm, and then cultured with Akt stimulator agents to activate the PI3K/Akt/FOXO3a and disrupt the Hippo signaling pathways [164]. As reported, PFs in POI patients hardly activated spontaneously [165], and thus this procedure is indicated to activate the residual PFs and promote follicle growth in patients with POI or low ovarian reserve [166]. Successful healthy live births are reported in both translational and clinical studies [167,168]. Until now, 177 patients have undergone IVA and have obtained accumulatively 26 pregnancies with 18 full-term babies. Of note, these live births could be obtained in POI patients with undetectable AMH and long-term menstruation [169]. This suggested that IVA should be beneficial for CCS patients exposed to chemo/radiotherapy who have not undergone any types of FP. Moreover, it is suggested that a combination of IVA with in vitro grow (IVG) follicles could develop a complete culture of PFs to obtain mature follicles in vitro, as discussed below.

### 4.4. Other Experimental Options

To eliminate the risks of reintroducing malignancy in OTC, the use of in vitro methods to obtain a component and mature oocyte from the small PFs has been recently challenged [170]. Another purpose of this approach is to maximize the number of mature oocytes from the obtained ovarian tissue to increase the likelihood of pregnancy. Healthy offspring are achieved in animal models [171]. Moreover, the first human MII oocyte has been achieved from early secondary human follicles [172,173]. However, this approach is still in the experimental stage. Since each stage of follicle development requires different conditions and involves growth factors, a dynamic culture system is mandatory to achieve follicles with fully developed oocytes from PFs [174]. Maintaining interactions between oocytes and surrounding GCs also needs to be addressed [43]. In addition, low oocyte maturation rate and reported morphological abnormalities in the matured oocytes are current inquiries that need to be addressed [170].

Another suggested strategy is generating in vitro oocytes from stem cells. It was reported that oogonial stem cells (OSCs) were successfully isolated from ovarian stem cells (OSCs) from animal and human ovaries [175]. In subsequent studies, OSCs obtained from mice could differentiate into fertilizable oocytes in vitro, resulting in embryo development [176]. Induced pluripotent stem cells and embryonic stem cells are other suggested sources for generating oocytes. In mice, competent oocytes resulting in healthy pups have been achieved from pluripotent stem cells [177]. In consistency, recent work reported similar success with embryonic stem cells [178]. These findings provided the scientific conceptions for developing stem cell approaches to produce new oocytes. This option is especially beneficial for CCS without residual follicles due to chemo- or radiation-therapy. Nonetheless, the existence and function OSCs in human FP still faces numerous debates [179,180]. Since the success has been reported uniquely in the mouse model, developing human oocytes from stem cells is still far from clinical implementation [8].

Another approach is growing PFs isolated from cryopreserved ovarian tissue and assembled on a 3D matrix to form an “artificial ovary” [181,182,183]. This matrix design allows for essential nutrient diffusion, follicular expansion, and resultant follicular growth [184,185]. The suggested scaffold matrixes are fibrin clots to decellularized ovaries and 3D-printed biopolymer networks [186,187,188,189]. In a mouse model experiment, the success of growing ovarian follicles on a bioplotted scaffold has been reported [182]. The vascularization was also observed in fibrin clots containing grafted ovarian cells [190]. In addition, an artificial ovary created by alginate microcapsules containing granulosa and theca cells could manifest the endocrine function [191], and primary ovarian cells seeded onto decellularized scaffolds successfully produced estradiol [192]. These results indicate that artificial ovaries can be a beneficial alternative option for both fertility expansion and endocrine function.

## 5. Protective Approaches to Ovarian Reserve during Chemo- and Radio-Therapy

In addition to above options of FPs, developing protective adjuvants to prevent follicle damage during chemotherapy is advantageous for oncological patients, especially for young ones [193,194,195]. Many agents have been proposed based on the current understandings of mechanisms of how chemo- and radio-therapy impact ovarian reserves.

Preclinical studies in mice, nonhuman primates, and humans have been performed and demonstrated with positive results [13,194,196,197].

### 5.1. The Fertoprotective Agents Preventing Follicular Apoptosis

The first agent in this group is sphingosine-1-phosphate (S1P), an inhibitor of sphingomyelinase which is one major protein inducing cell apoptosis. The major activity of S1P is acting as an intracellular second messenger inhibiting apoptosis pathways, promoting angiogenesis and cell migration [198,199]. S1P pretreatment protected human ovarian tissue from damage induced by cyclophosphamide and doxorubicin [40]. Treatment with S1P shows protective effects against damage of dacarbazin in mouse ovaries [200], and of cyclophosphamide and doxorubicin in human ovaries [40]. In macaques, live births are achieved after cotreatment of S1P with radiation exposure [201]. However, implementation of S1P in the clinical setting is still challenging, because S1P must be administered directly into the ovary.

The second agent is imatinib, an inhibitor of c-ABI kinase which is one component in the apoptotic pathway of PFs. Imatinib has been proposed to suppress the cisplatin-induced follicular loss by DNA damage [41,202]. In a later experiment, imatinib cotreatment with cisplatin reduces the percentage of unhealthy follicles by 21% [58].

Furthermore, several other agents are suggested to prevent the DNA damage induced by chemotherapy, leading to reduction in follicular apoptosis. For instance, dexrazoxane prevents the DNA damage and the activation of gamma-H2AX induced by doxorubicin in mouse and marmoset ovarian tissues [203,204]. KU55933, an inhibitor of ataxia–telangiectasia mutated (ATM) protein, known to regulate the response to DNA damage, protects follicles at all stages in mouse ovaries from apoptosis induced by ATM activation [205]. Inhibition of ataxia telangiectasia and Rad3 related protein, another fundamental regulator of DNA damage checkpoints, also protects follicles at all stages in mouse ovaries from apoptosis induced by cyclophosphamide [46]. Melatonin is also reported to have a protective effect on the regulation of DNA damage response and repair [206].

Another molecule is tamoxifen, which is suggested to upregulate insulin-like growth factor 1 and protect the PFs from oxidative stress [66]. In mice, the cotreatment of tamoxifen with cyclophosphamide and doxorubicin preserves the ovarian reserve [207]. The co-treatment of ceramide-1-phosphate with cyclophosphamide also decreases the deleterious effect of cyclophosphamide in mice ovaries [13].

### 5.2. The Fertoprotective Agent Preventing Accelerated PFs’ Activation

Based on the understanding of PF activation induced by chemotherapeutic agents via the PI3K/Akt/FOXO3a pathway, several studies have suggested the usage of mTOR1 and mTOCR1/2 inhibitors to preserve ovarian reserve [60,65,208]. Rapamycin, an inhibitor of mTOCR1, also prevents the cyclophosphamide-induced PFs’ activation [65,209]. Melatonin interferes with PF’s activation caused by cisplatin through mediating PTEN and inhibiting Akt, glycogen synthase kinase 3 beta, and FOXO3 activation in mice [210].

Another molecule is ammonium trichloro (dioxoethylene-o,o′) tellurate (AS101), a nontoxic immunomodulatory compound, which regulates the PI3K–Pten–Akt pathway [211]. In a mouse model, AS101 inhibits the activation of the PI3K pathway induced by cyclophosphamide, resulting in prevention of PFs activation [211].

In addition, as indicated to inhibit the PFs’ activation [164], AMH is also a potential agent. In mice, AMH cotreatment with chemotherapy is reported to limit the PF activation triggered by cyclophosphamide, doxorubicin, or cisplatin [212]. The protective effect of recombinant AMH is also presented in mouse experiments, as pharmacological administration of recombinant human AMH during chemotherapy in mice reduces the activation of PFs by suppression of the PI3K signaling pathway and preserves fertility [68].

GnRH agonists have been used to protect the ovaries during chemotherapy in recent decades [213,214]. Several clinical data indicate the positive results supporting the use of GnRH agonists as protective agents during chemotherapy. In a meta-analysis including 873 breast cancer patients, the administration of GnRH agonist during chemotherapy reduces the POI induction (from 30.9 to 14.1%) and increases the number of post-treatment pregnancies [215]. However, the efficacy of GnRH agonist in ovarian protection still faces several objections because of the lack of a support mechanism [216]. According to the American Society of Clinical Oncology 2018, EHSRE 2020, and the European Society for Medical Oncology 2020, existing data are not sufficient to support the implementation of GnRH agonist as a fertoprotective agent [87,215,217].

### 5.3. Fertoprotective Agents Improving Vascularization

Regarding the vascular damage provoked by chemotherapy, granulocyte colony-stimulating factor (G-CSF) is supposed to ameliorate vascularization after chemotherapy. The mechanisms of how G-CSF improves vascularization are still not elucidated. It is suggested that G-CSF upregulates intracellular adhesion molecule 1, enhancing the migration of monocytes which engage in angiogenesis in the local tissue [218]. In mice, cotreatment of G-CSF during chemotherapy using cyclophosphamide or busulfan increases microvessel density, resulting in a reduction in the follicular loss and prolonging the time to POI [219]. In consistency, mice treated with both cisplatin and G-CSF have a higher follicle count and higher serum AMH levels in comparison with mice exposed to cisplatin alone [220].

### 5.4. Ovarian Transposition during Radiotherapy

In the case of pelvic radiation, ovarian transposition is suggested to protect the ovary during radiation [214,221]. Accordingly, patients’ ovaries are mobilized with their vascular pedicle and transposed to another position marked with radio-opaque clips for later identification. This is usually indicated for patients receiving pelvic radiotherapy for cervical cancer, vaginal, rectal, or anal cancers, pelvic lymphoma or Ewing’s sarcoma [215,222]. In recent meta-analysis, the mean rate of ovarian function recovered in patients who had ovarian transposition before pelvic radiotherapy was 61.7% (431/699) [223].

## 6. Future Perspectives of FP

Although substantial advances in FP have been yielded, encouraging achievements for modern oncofertility, ongoing efforts are still essential to address existing challenges, including developing approaches to eliminate the risk of cancer reintroduction in OTC, clarifying genomics markers to predict the likelihood of achieving pregnancy, optimizing the novel therapies (IVA and IVM), further investigating several experimental options (IVG, artificial ovary, and stem cell therapy), and promoting the implementation of protective options for patients prior to gonadotoxic treatment.

Advances in genomic science that could facilitate oncofertility are also encouraging achievements. Some works have tried to develop a system of genetic markers to predict the susceptibility to chemotherapy-induced damage [19]. Genetic mutations involving the DNA repairing system are supposed factors increasing ovarian susceptibility to chemotherapy-induced damage [19]. Several genetic defects in *BRCA1/2* [224,225], *minichromosome maintenance complex component 8/9* [226], *helicase for meiosis 1* [227], *nucleoporin 107* [228], and *synaptonemal complex central element protein 1* [229] are suggested to be implicated in ovarian insufficiency. Disparities in steroid production and altered expression of specific microRNAs (miRNAs), e.g., miRNA–193b, miRNA–320A, and miRNA–24, after cryopreservation are also related to impaired ovarian functioning and folliculogenesis [230]. For OTC, recent advances allow examination of tissue vitality; structural, functional, and DNA integrity; malignant cell contamination; and detection of molecular tumor markers [231]. One study reported that the implementation of a new miRNA array test in the slow-freezing protocol could yield a rate of human ovarian tissue with no structural damage as high as 80% [232].

Although the development of fertoprotective agents has risen recently, the data in transitional and clinical studies are still limited. In addition, there is still no proposed agent to ameliorate the oocyte quality after chemotherapeutic agents or radiation exposure. In addition, improvement of the social consideration of FP is another inquiry. Despite being implemented for nearly two decades, FP is currently not a routine clinical practice. Healthcare providers rarely consult appropriate information about FP to cancer patients in developing countries [233,234]. In developed countries, it is reported that only 17.5% of otorhinolaryngologists consulted FP to their young cancer patients before undergoing chemotherapy [235]. In addition, the financial barrier is another factor inferring the accessibility of FP for cancer patients [236].

## 7. Conclusions

In summary, antineoplastic agents and radiation cause extensive follicle impairment in both quantity and quality aspects. Since the first initial studies, FP with a variety of options has yielded hundreds of live births for CCS. The mechanisms of ovarian dysfunction induced by chemo- and radio-therapy have been gradually clarified to support the development of fertoprotective agents. However, several inquiries remain to be addressed to increase the effectiveness as well as to ensure the safety of FP.

## Figures and Tables

**Table 1:** Summary of published clinical studies describing chemo- and radio-therapy on ovarian function.

Authors	Number of CCS	Age	Exposure Agent ^a^	Radiation	Effects	
					Clinical	Laboratory Test
Berjeb et al. (2021) [20]	66	15–40 (26.7 ± 6.8)	Bleomycin, etoposide, doxorubicin, cyclophosphamide, vincristine, procarbazine, doxorubicin, vinblastine, dacarbazine	No	N/A	↓ AMH
Filippi et al. (2021) [21]	90	21.3 ± 5.4	Bleomycin, cisplatin, bleomycin, dacarbazine-vinblastine	Yes/No ^b^	↑ POI rate (21% of treated women)	
Gini et al. (2019) [22]	97	16–50 (median: 28)	Doxorubicin, cyclophosphamide, vincristine, bleomycin	Yes	↑ Amenorrhea	N/A
Lehmann et al. (2019) [23]	444	≤40	N/A	Yes/No	N/A	↑ LH ↑ FSH ↓ E2
Anderson et al. (2018) [4]	23,201	≤39	N/A	N/A	↓ Pregnancy rate (↓ 38%)	
Shandley et al. (2018) [24]	1090	20–35 (median: 26)	N/A	No	N/A	↓ AFC↓ AMH
Sinha et al. (2018) [25]	88	24–43 (median: 35)	Taxotere, cyclophosphamide, carboplatin, fluorouracil, epirubicin	No	N/A	↓ AFC
Al-Rawi et al. (2018) [26]	58	25–45 (38.83 ± 4.74)	Anthracycline, cyclophosphamide	No	N/A	↓ AFC↓ E2 ↑ LH
Aderson et al. (2018) [27]	67	18–45	Doxorubicin, bleomycin, vinblastine, and dacarbazine	No	N/A	↓ AMH ↑ FSH ↓ E2
Levine et al. (2018) [28]	2930	18–58 (median: 32)	Alkylating agent, procarbazine	Yes/No	↑ POI rate (9.1% of treated women)	N/A
Armuand et al. (2017) [29]	552	≥13	N/A	N/A	↓ The probability of having a first live birth	N/A
Chemaitilly et al. (2017) [30]	988	18–45 (median: 31.7)	Alkylating agents	Yes	↑ POI rate (10.9% of treated women)	N/A
D’Avila et al. (2017) [31]	52	27–40 (35.3 ± 3.8)	Cyclophosphamide	No	↑ Amenorrhea	↓ AFC↓ AMH ↑ FSH
Abir et al. (2016) [32]	20	5–18	Alkylating agents, bleomycin, cisplatin, vincristine, etoposide, carboplatin, doxorubicin, etopside, doxorubicin, bleomycin, vinblastine, dacarbazine.	No	↑ Atretic follicles↓ Oocyte maturation	N/A
Hamy et al. (2016) [33]	134	26–43 (median: 36)	Anthracyclines, taxane	No	N/A	↓ AMH
Even-Or et al. (2016) [34]	35	13–36 (median: 25.5)	Melphalan	No	N/A	↓ AMH ↑ FSH ↓ LH
Gupta et al. (2016) [35]	16	11–18 (median: 14.3)	Doxorubicin, cyclophosphamide, cisplatin	No	↑ Amenorrhea	↓ AMH
Chow et al. (2016) [5]	5298	15–44	Busulfan, carboplatin, carmustine, chlorambucil, chlormethine, cisplatin, cyclophosphamide, dacarbazine, ifosfamide, lomustine, melphalan, procarbazine, temozolomide	Yes/No	↓ Pregnancy rate	N/A
Thomas-Teinturier et al. (2015) [36]	105	18–39 (median: 21.5)	Cyclophosphamide, ifosfamide	Yes	N/A	↓AMH ↑ FSH
Behringer et al. (2012) [37]	106	18–39 (28 ± 7)	Bleomycin, etoposide, doxorubicin, cyclophosphamide, vincristine, procarbazine, doxorubicin, bleomycin, vinblastine, dacarbazine	N/A	N/A	↓ AMH ↑ FSH
Green et al. (2009) [38]	5149	15–44	Alkylating agents	Yes/No	↓ Pregnancy rate	N/A

^a^: All chemotherapeutic agents exposed that all included patients were exposed to are listed in each study. ^b^: Some patients treated by both radiation and chemotherapy, but some patients were treated only with chemotherapy. ↓: Decreased. ↑: Increased. AFC: antral follicle count, AMH: anti-Müllerian hormone, E2: estradiol, FSH: follicle-stimulating hormone, LH: luteinizing hormone, N/A: not available or not applicable, POI: premature ovarian insufficiency.

## References

[1] Siegel R.L., Miller K.D., Jemal A. (2019). Cancer statistics, 2019. CA Cancer J. Clin..

[2] Trama A., Bernasconi A., McCabe M.G., Guevara M., Gatta G., Botta L., Ries L., Bleyer A. (2019). Is the cancer survival improvement in European and American adolescent and young adults still lagging behind that in children?. Pediatr. Blood Cancer.

[3] Trama A., Botta L., Foschi R., Ferrari A., Stiller C., Desandes E., Maule M.M., Merletti F., Gatta G. (2016). Survival of European adolescents and young adults diagnosed with cancer in 2000–07: Population-based data from EUROCARE-5. Lancet Oncol..

[4] Anderson R.A., Brewster D.H., Wood R., Nowell S., Fischbacher C., Kelsey T.W., Wallace W.H.B. (2018). The impact of cancer on subsequent chance of pregnancy: A population-based analysis. Hum. Reprod..

[5] Chow E.J., Stratton K.L., Leisenring W.M., Oeffinger K.C., Sklar C.A., Donaldson S.S., Ginsberg J.P., Kenney L.B., Levine J.M., Robison L.L. (2016). Pregnancy after chemotherapy in male and female survivors of childhood cancer treated between 1970 and 1999: A report from the Childhood Cancer Survivor Study cohort. Lancet Oncol..

[6] Overbeek A., van den Berg M.H., van Leeuwen F.E., Kaspers G.J., Lambalk C.B., van Dulmen-den Broeder E. (2017). Chemotherapy-related late adverse effects on ovarian function in female survivors of childhood and young adult cancer: A systematic review. Cancer Treat. Rev..

[7] Anderson R.A., Mitchell R.T., Kelsey T.W., Spears N., Telfer E.E., Wallace W.H. (2015). Cancer treatment and gonadal function: Experimental and established strategies for fertility preservation in children and young adults. Lancet Diabetes Endocrinol..

[8] Pampanini V., Hassan J., Oliver E., Stukenborg J.B., Damdimopoulou P., Jahnukainen K. (2020). Fertility Preservation for Prepubertal Patients at Risk of Infertility: Present Status and Future Perspectives. Horm. Res. Paediatr..

[9] Morgan S., Anderson R.A., Gourley C., Wallace W.H., Spears N. (2012). How do chemotherapeutic agents damage the ovary?. Hum. Reprod. Update.

[10] Sonigo C., Beau I., Binart N., Grynberg M. (2019). The Impact of Chemotherapy on the Ovaries: Molecular Aspects and the Prevention of Ovarian Damage. Int. J. Mol. Sci..

[11] Szymanska K.J., Tan X., Oktay K. (2020). Unraveling the mechanisms of chemotherapy-induced damage to human primordial follicle reserve: Road to developing therapeutics for fertility preservation and reversing ovarian aging. Mol. Hum. Reprod..

[12] Yuksel A., Bildik G., Senbabaoglu F., Akin N., Arvas M., Unal F., Kilic Y., Karanfil I., Eryılmaz B., Yilmaz P. (2015). The magnitude of gonadotoxicity of chemotherapy drugs on ovarian follicles and granulosa cells varies depending upon the category of the drugs and the type of granulosa cells. Hum. Reprod..

[13] Pascuali N., Scotti L., Di Pietro M., Oubiña G., Bas D., May M., Muñoz A.G., Cuasnicú P.S., Cohen D.J., Tesone M. (2018). Ceramide-1-phosphate has protective properties against cyclophosphamide-induced ovarian damage in a mice model of premature ovarian failure. Hum. Reprod..

[14] Wallace W.H., Thomson A.B., Saran F., Kelsey T.W. (2005). Predicting age of ovarian failure after radiation to a field that includes the ovaries. Int. J. Radiat Oncol. Biol. Phys..

[15] Duffy C., Allen S. (2009). Medical and psychosocial aspects of fertility after cancer. Cancer J..

[16] Niedzwiedz C.L., Knifton L., Robb K.A., Katikireddi S.V., Smith D.J. (2019). Depression and anxiety among people living with and beyond cancer: A growing clinical and research priority. BMC Cancer.

[17] Howard-Anderson J., Ganz P.A., Bower J.E., Stanton A.L. (2012). Quality of life, fertility concerns, and behavioral health outcomes in younger breast cancer survivors: A systematic review. J. Natl. Cancer Inst..

[18] Logan S., Perz J., Ussher J.M., Peate M., Anazodo A. (2019). Systematic review of fertility-related psychological distress in cancer patients: Informing on an improved model of care. Psychooncology.

[19] Sun B., Yeh J. (2021). Onco-fertility and personalized testing for potential for loss of ovarian reserve in patients undergoing chemotherapy: Proposed next steps for development of genetic testing to predict changes in ovarian reserve. Fertil. Res. Pract..

[20] Berjeb K.K., Debbabi L., Braham M., Zemni Z., Chtourou S., Hannachi H., Hamdoun M., Ayadi M., Kacem K., Zhioua F. (2021). Evaluation of ovarian reserve before and after chemotherapy. J. Gynecol. Obstet. Hum. Reprod..

[21] Filippi F., Meazza C., Somigliana E., Podda M., Dallagiovanna C., Massimino M., Raspagliesi F., Terenziani M. (2021). Fertility preservation in childhood and adolescent female tumor survivors. Fertil. Steril..

[22] Gini G., Annibali O., Lupasco D., Bocci C., Tomarchio V., Sampaolo M., Trappolini S., Tafuri M.A., Cacciagiù S., Ciccarone M. (2019). Gonadal Function Recovery and Fertility in Women Treated with Chemo- and/or Radiotherapy for Hodgkin’s and Non-Hodgkin Lymphoma. Chemotherapy.

[23] Lehmann V., Chemaitilly W., Lu L., Green D.M., Kutteh W.H., Brinkman T.M., Srivastava D.K., Robison L.L., Hudson M.M., Klosky J.L. (2019). Gonadal Functioning and Perceptions of Infertility Risk Among Adult Survivors of Childhood Cancer: A Report From the St Jude Lifetime Cohort Study. J. Clin. Oncol..

[24] Shandley L.M., Fothergill A., Spencer J.B., Mertens A.C., Cottrell H.N., Howards P.P. (2018). Impact of cancer treatment on risk of infertility and diminished ovarian reserve in women with polycystic ovary syndrome. Fertil. Steril..

[25] Sinha N., Letourneau J.M., Wald K., Xiong P., Imbar T., Li B., Harris E., Mok-Lin E., Cedars M.I., Rosen M.P. (2018). Antral follicle count recovery in women with menses after treatment with and without gonadotropin-releasing hormone agonist use during chemotherapy for breast cancer. J. Assist. Reprod. Genet..

[26] Al-Rawi S.A., Saleh B.O., Al-Naqqash M.A. (2018). Serum anti-müllerian hormone levels in evaluation of chemotherapy effect on ovarian reserve in women with breast cancer. A follow-up study. Saudi Med. J..

[27] Anderson R.A., Remedios R., Kirkwood A.A., Patrick P., Stevens L., Clifton-Hadley L., Roberts T., Hatton C., Kalakonda N., Milligan D.W. (2018). Determinants of ovarian function after response-adapted therapy in patients with advanced Hodgkin’s lymphoma (RATHL): A secondary analysis of a randomised phase 3 trial. Lancet Oncol..

[28] Levine J.M., Whitton J.A., Ginsberg J.P., Green D.M., Leisenring W.M., Stovall M., Robison L.L., Armstrong G.T., Sklar C.A. (2018). Nonsurgical premature menopause and reproductive implications in survivors of childhood cancer: A report from the Childhood Cancer Survivor Study. Cancer.

[29] Armuand G., Skoog-Svanberg A., Bladh M., Sydsjö G. (2017). Reproductive Patterns Among Childhood and Adolescent Cancer Survivors in Sweden: A Population-Based Matched-Cohort Study. J. Clin. Oncol..

[30] Chemaitilly W., Li Z., Krasin M.J., Brooke R.J., Wilson C.L., Green D.M., Klosky J.L., Barnes N., Clark K.L., Farr J.B. (2017). Premature Ovarian Insufficiency in Childhood Cancer Survivors: A Report From the St. Jude Lifetime Cohort. J. Clin. Endocrinol. Metab..

[31] D’Avila Â.M., Capp E., Corleta H.V.E. (2017). Antral Follicles Count and Anti-Müllerian Hormone Levels after Gonadotoxic Chemotherapy in Patients with Breast Cancer: Cohort Study. Rev. Bras. Ginecol. Obstet..

[32] Abir R., Ben-Aharon I., Garor R., Yaniv I., Ash S., Stemmer S.M., Ben-Haroush A., Freud E., Kravarusic D., Sapir O. (2016). Cryopreservation of in vitro matured oocytes in addition to ovarian tissue freezing for fertility preservation in paediatric female cancer patients before and after cancer therapy. Hum. Reprod..

[33] Hamy A.S., Porcher R., Eskenazi S., Cuvier C., Giacchetti S., Coussy F., Hocini H., Tournant B., Perret F., Bonfils S. (2016). Anti-Müllerian hormone in breast cancer patients treated with chemotherapy: A retrospective evaluation of subsequent pregnancies. Reprod. Biomed. Online.

[34] Even-Or E., Ben-Haroush A., Yahel A., Yaniv I., Stein J. (2016). Fertility After Treatment With High Dose Melphalan in Women With Acute Myelogenous Leukemia. Pediatr. Blood Cancer.

[35] Gupta A.A., Chong A.L., Deveault C., Traubici J., Maloney A.M., Knight S., Lorenzo A., Allen L. (2016). Anti-Müllerian Hormone in Female Adolescent Cancer Patients Before, During, and After Completion of Therapy: A Pilot Feasibility Study. J. Pediatr. Adolesc. Gynecol..

[36] Thomas-Teinturier C., Allodji R.S., Svetlova E., Frey M.A., Oberlin O., Millischer A.E., Epelboin S., Decanter C., Pacquement H., Tabone M.D. (2015). Ovarian reserve after treatment with alkylating agents during childhood. Hum. Reprod..

[37] Behringer K., Thielen I., Mueller H., Goergen H., Eibl A.D., Rosenbrock J., Halbsguth T., Eichenauer D.A., Fuchs M., Reiners K.S. (2012). Fertility and gonadal function in female survivors after treatment of early unfavorable Hodgkin lymphoma (HL) within the German Hodgkin Study Group HD14 trial. Ann. Oncol..

[38] Green D.M., Kawashima T., Stovall M., Leisenring W., Sklar C.A., Mertens A.C., Donaldson S.S., Byrne J., Robison L.L. (2009). Fertility of female survivors of childhood cancer: A report from the childhood cancer survivor study. J. Clin. Oncol..

[39] Soleimani R., Heytens E., Darzynkiewicz Z., Oktay K. (2011). Mechanisms of chemotherapy-induced human ovarian aging: Double strand DNA breaks and microvascular compromise. Aging.

[40] Li F., Turan V., Lierman S., Cuvelier C., De Sutter P., Oktay K. (2014). Sphingosine-1-phosphate prevents chemotherapy-induced human primordial follicle death. Hum. Reprod..

[41] Gonfloni S., Di Tella L., Caldarola S., Cannata S.M., Klinger F.G., Di Bartolomeo C., Mattei M., Candi E., De Felici M., Melino G. (2009). Inhibition of the c-Abl-TAp63 pathway protects mouse oocytes from chemotherapy-induced death. Nat. Med..

[42] Bedoschi G., Navarro P.A., Oktay K. (2016). Chemotherapy-induced damage to ovary: Mechanisms and clinical impact. Future Oncol..

[43] Donnez J., Dolmans M.M. (2017). Fertility Preservation in Women. N. Engl. J. Med..

[44] Lambertini M., Olympios N., Lequesne J., Calbrix C., Fontanilles M., Loeb A., Leheurteur M., Demeestere I., Di Fiore F., Perdrix A. (2019). Impact of Taxanes, Endocrine Therapy, and Deleterious Germline BRCA Mutations on Anti-müllerian Hormone Levels in Early Breast Cancer Patients Treated With Anthracycline- and Cyclophosphamide-Based Chemotherapy. Front. Oncol..

[45] Dumontet C., Jordan M.A. (2010). Microtubule-binding agents: A dynamic field of cancer therapeutics. Nat. Rev. Drug Discov..

[46] Luan Y., Edmonds M.E., Woodruff T.K., Kim S.Y. (2019). Inhibitors of apoptosis protect the ovarian reserve from cyclophosphamide. J. Endocrinol..

[47] Jeelani R., Khan S.N., Shaeib F., Kohan-Ghadr H.R., Aldhaheri S.R., Najafi T., Thakur M., Morris R., Abu-Soud H.M. (2017). Cyclophosphamide and acrolein induced oxidative stress leading to deterioration of metaphase II mouse oocyte quality. Free Radic. Biol. Med..

[48] Kerr J.B., Hutt K.J., Michalak E.M., Cook M., Vandenberg C.J., Liew S.H., Bouillet P., Mills A., Scott C.L., Findlay J.K. (2012). DNA damage-induced primordial follicle oocyte apoptosis and loss of fertility require TAp63-mediated induction of Puma and Noxa. Mol. Cell.

[49] Winship A.L., Stringer J.M., Liew S.H., Hutt K.J. (2018). The importance of DNA repair for maintaining oocyte quality in response to anti-cancer treatments, environmental toxins and maternal ageing. Hum. Reprod. Update.

[50] Nguyen Q.N., Zerafa N., Liew S.H., Findlay J.K., Hickey M., Hutt K.J. (2019). Cisplatin- and cyclophosphamide-induced primordial follicle depletion is caused by direct damage to oocytes. Mol. Hum. Reprod..

[51] Nguyen Q.N., Zerafa N., Liew S.H., Morgan F.H., Strasser A., Scott C.L., Findlay J.K., Hickey M., Hutt K.J. (2018). Loss of PUMA protects the ovarian reserve during DNA-damaging chemotherapy and preserves fertility. Cell Death Dis..

[52] Oktem O., Oktay K. (2007). A novel ovarian xenografting model to characterize the impact of chemotherapy agents on human primordial follicle reserve. Cancer Res..

[53] Bildik G., Acılan C., Sahin G.N., Karahuseyinoglu S., Oktem O. (2018). C-Abl is not actıvated in DNA damage-induced and Tap63-mediated oocyte apoptosıs in human ovary. Cell Death Dis..

[54] Kalich-Philosoph L., Roness H., Carmely A., Fishel-Bartal M., Ligumsky H., Paglin S., Wolf I., Kanety H., Sredni B., Meirow D. (2013). Cyclophosphamide triggers follicle activation and “burnout”; AS101 prevents follicle loss and preserves fertility. Sci. Transl. Med..

[55] Xie Y., Li S., Zhou L., Lin H., Jiao X., Qiu Q., Liang Y., Zhang Q. (2020). Rapamycin preserves the primordial follicle pool during cisplatin treatment in vitro and in vivo. Mol. Reprod. Dev..

[56] Rossi V., Lispi M., Longobardi S., Mattei M., Di Rella F., Salustri A., De Felici M., Klinger F.G. (2017). LH prevents cisplatin-induced apoptosis in oocytes and preserves female fertility in mouse. Cell Death Differ..

[57] Allen C.M., Lopes F., Mitchell R.T., Spears N. (2020). Comparative gonadotoxicity of the chemotherapy drugs cisplatin and carboplatin on prepubertal mouse gonads. Mol. Hum. Reprod..

[58] Morgan S., Lopes F., Gourley C., Anderson R.A., Spears N. (2013). Cisplatin and doxorubicin induce distinct mechanisms of ovarian follicle loss; imatinib provides selective protection only against cisplatin. PLoS ONE.

[59] Chang E.M., Lim E., Yoon S., Jeong K., Bae S., Lee D.R., Yoon T.K., Choi Y., Lee W.S. (2015). Cisplatin Induces Overactivation of the Dormant Primordial Follicle through PTEN/AKT/FOXO3a Pathway which Leads to Loss of Ovarian Reserve in Mice. PLoS ONE.

[60] Goldman K.N., Chenette D., Arju R., Duncan F.E., Keefe D.L., Grifo J.A., Schneider R.J. (2017). mTORC1/2 inhibition preserves ovarian function and fertility during genotoxic chemotherapy. Proc. Natl. Acad. Sci. USA.

[61] Lande Y., Fisch B., Tsur A., Farhi J., Prag-Rosenberg R., Ben-Haroush A., Kessler-Icekson G., Zahalka M.A., Ludeman S.M., Abir R. (2017). Short-term exposure of human ovarian follicles to cyclophosphamide metabolites seems to promote follicular activation in vitro. Reprod. Biomed. Online.

[62] Chen X.Y., Xia H.X., Guan H.Y., Li B., Zhang W. (2016). Follicle Loss and Apoptosis in Cyclophosphamide-Treated Mice: What’s the Matter?. Int. J. Mol. Sci..

[63] Wang Y., Liu M., Johnson S.B., Yuan G., Arriba A.K., Zubizarreta M.E., Chatterjee S., Nagarkatti M., Nagarkatti P., Xiao S. (2019). Doxorubicin obliterates mouse ovarian reserve through both primordial follicle atresia and overactivation. Toxicol. Appl. Pharmacol..

[64] Shai D., Aviel-Ronen S., Spector I., Raanani H., Shapira M., Gat I., Roness H., Meirow D. (2021). Ovaries of patients recently treated with alkylating agent chemotherapy indicate the presence of acute follicle activation, elucidating its role among other proposed mechanisms of follicle loss. Fertil. Steril..

[65] Zhou L., Xie Y., Li S., Liang Y., Qiu Q., Lin H., Zhang Q. (2017). Rapamycin Prevents cyclophosphamide-induced Over-activation of Primordial Follicle pool through PI3K/Akt/mTOR Signaling Pathway in vivo. J. Ovarian Res..

[66] Roness H., Kashi O., Meirow D. (2016). Prevention of chemotherapy-induced ovarian damage. Fertil. Steril..

[67] Jang H., Hong K., Choi Y. (2017). Melatonin and Fertoprotective Adjuvants: Prevention against Premature Ovarian Failure during Chemotherapy. Int. J. Mol. Sci..

[68] Roness H., Spector I., Leichtmann-Bardoogo Y., Savino A.M., Dereh-Haim S., Meirow D. (2019). Pharmacological administration of recombinant human AMH rescues ovarian reserve and preserves fertility in a mouse model of chemotherapy, without interfering with anti-tumoural effects. J. Assist. Reprod. Genet..

[69] Titus S., Szymanska K.J., Musul B., Turan V., Taylan E., Garcia-Milian R., Mehta S., Oktay K. (2021). Individual-oocyte transcriptomic analysis shows that genotoxic chemotherapy depletes human primordial follicle reserve in vivo by triggering proapoptotic pathways without growth activation. Sci. Rep..

[70] Meirow D., Epstein M., Lewis H., Nugent D., Gosden R.G. (2001). Administration of cyclophosphamide at different stages of follicular maturation in mice: Effects on reproductive performance and fetal malformations. Hum. Reprod..

[71] Meirow D., Nugent D. (2001). The effects of radiotherapy and chemotherapy on female reproduction. Hum. Reprod. Update.

[72] Luo Q., Yin N., Zhang L., Yuan W., Zhao W., Luan X., Zhang H. (2017). Role of SDF-1/CXCR4 and cytokines in the development of ovary injury in chemotherapy drug induced premature ovarian failure mice. Life Sci..

[73] Meirow D., Dor J., Kaufman B., Shrim A., Rabinovici J., Schiff E., Raanani H., Levron J., Fridman E. (2007). Cortical fibrosis and blood-vessels damage in human ovaries exposed to chemotherapy. Potential mechanisms of ovarian injury. Hum. Reprod..

[74] Bar-Joseph H., Ben-Aharon I., Tzabari M., Tsarfaty G., Stemmer S.M., Shalgi R. (2011). In vivo bioimaging as a novel strategy to detect doxorubicin-induced damage to gonadal blood vessels. PLoS ONE.

[75] Oktem O., Oktay K. (2007). Quantitative assessment of the impact of chemotherapy on ovarian follicle reserve and stromal function. Cancer.

[76] Wallace W.H., Thomson A.B., Kelsey T.W. (2003). The radiosensitivity of the human oocyte. Hum. Reprod..

[77] Meirow D., Biederman H., Anderson R.A., Wallace W.H. (2010). Toxicity of chemotherapy and radiation on female reproduction. Clin. Obstet. Gynecol..

[78] Wo J.Y., Viswanathan A.N. (2009). Impact of radiotherapy on fertility, pregnancy, and neonatal outcomes in female cancer patients. Int. J. Radiat. Oncol. Biol. Phys..

[79] Ginsberg J.P. (2011). New advances in fertility preservation for pediatric cancer patients. Curr. Opin. Pediatr..

[80] Dolmans M.M., von Wolff M., Poirot C., Diaz-Garcia C., Cacciottola L., Boissel N., Liebenthron J., Pellicer A., Donnez J., Andersen C.Y. (2021). Transplantation of cryopreserved ovarian tissue in a series of 285 women: A review of five leading European centers. Fertil. Steril..

[81] Yasmin E., Mitchell R., Lane S. (2021). Preservation of fertility in teenagers and young adults treated for haematological malignancies. Lancet Haematol..

[82] Abir R., Ben-Haroush A., Felz C., Okon E., Raanani H., Orvieto R., Nitke S., Fisch B. (2008). Selection of patients before and after anticancer treatment for ovarian cryopreservation. Hum. Reprod..

[83] Pligina K.L., Zhanataev A.K., Kulakova A.V., Daugel-Dauge N.O., Durnev A.D. (2017). Induced Aneugenic Effects in Mouse Oocytes In Vivo. Bull. Exp. Biol. Med..

[84] Fabbri R., Vicenti R., Macciocca M., Pasquinelli G., Lima M., Parazza I., Magnani V., Venturoli S. (2012). Cryopreservation of ovarian tissue in pediatric patients. Obstet. Gynecol. Int..

[85] Fabiani C., Ferrante M.G., Meneghini C., Licata E., Paciotti G., Gallo M., Schiavi M., Spina V., Guarino A., Caserta D. (2021). Female fertility preservation: Impact of cancer on ovarian function and oocyte quality. Int. J. Gynaecol. Obstet..

[86] Practice Committees of the American Society for Reproductive Medicine and the Society for Assisted Reproductive Technology (2013). Mature oocyte cryopreservation: A guideline. Fertil. Steril..

[87] Anderson R.A., Amant F., Braat D., D’Angelo A., de Sousa Lopes S.M.C., Demeestere I., Dwek S., Frith L., Lambertini M., Maslin C. (2020). ESHRE guideline: Female fertility preservation. Hum. Reprod. Open.

[88] Lee J.R., Choi Y.S., Jee B.C., Ku S.Y., Suh C.S., Kim K.C., Lee W.D., Kim S.H. (2007). Cryopreserved blastocyst transfer: Impact of gonadotropin-releasing hormone agonist versus antagonist in the previous oocyte retrieval cycles. Fertil. Steril..

[89] Eftekhar M., Firouzabadi R.D., Karimi H., Rahmani E. (2012). Outcome of cryopreserved-thawed embryo transfer in the GnRH agonist versus antagonist protocol. Iran. J. Reprod. Med..

[90] Checa M.A., Brassesco M., Sastre M., Gómez M., Herrero J., Marque L., Brassesco A., Espinós J.J. (2015). Random-start GnRH antagonist for emergency fertility preservation: A self-controlled trial. Int. J. Womens Health.

[91] Marklund A., Eloranta S., Wikander I., Kitlinski M.L., Lood M., Nedstrand E., Thurin-Kjellberg A., Zhang P., Bergh J., Rodriguez-Wallberg K.A. (2020). Efficacy and safety of controlled ovarian stimulation using GnRH antagonist protocols for emergency fertility preservation in young women with breast cancer-a prospective nationwide Swedish multicenter study. Hum. Reprod..

[92] Rodgers R.J., Reid G.D., Koch J., Deans R., Ledger W.L., Friedlander M., Gilchrist R.B., Walters K.A., Abbott J.A. (2017). The safety and efficacy of controlled ovarian hyperstimulation for fertility preservation in women with early breast cancer: A systematic review. Hum. Reprod..

[93] Rienzi L., Gracia C., Maggiulli R., LaBarbera A.R., Kaser D.J., Ubaldi F.M., Vanderpoel S., Racowsky C. (2017). Oocyte, embryo and blastocyst cryopreservation in ART: Systematic review and meta-analysis comparing slow-freezing versus vitrification to produce evidence for the development of global guidance. Hum. Reprod. Update.

[94] AbdelHafez F.F., Desai N., Abou-Setta A.M., Falcone T., Goldfarb J. (2010). Slow freezing, vitrification and ultra-rapid freezing of human embryos: A systematic review and meta-analysis. Reprod. Biomed. Online.

[95] Debrock S., Peeraer K., Gallardo E.F., De Neubourg D., Spiessens C., D’Hooghe T.M. (2015). Vitrification of cleavage stage day 3 embryos results in higher live birth rates than conventional slow freezing: A RCT. Hum. Reprod..

[96] Alexander V.M., Martin C.E., Schelble A.P., Laufer A.B., Hardi A., McKenzie L.J., Hipp H.S., Kawwass J.F., Spencer J.B., Jungheim E.S. (2021). Ovarian stimulation for fertility preservation in women with cancer: A systematic review and meta-analysis comparing random and conventional starts. J. Gynecol. Obstet. Hum. Reprod..

[97] Bonardi B., Massarotti C., Bruzzone M., Goldrat O., Mangili G., Anserini P., Spinaci S., Arecco L., Del Mastro L., Ceppi M. (2020). Efficacy and Safety of Controlled Ovarian Stimulation With or Without Letrozole Co-administration for Fertility Preservation: A Systematic Review and Meta-Analysis. Front. Oncol..

[98] Kim J., Turan V., Oktay K. (2016). Long-Term Safety of Letrozole and Gonadotropin Stimulation for Fertility Preservation in Women With Breast Cancer. J. Clin. Endocrinol. Metab..

[99] Maman E., Meirow D., Brengauz M., Raanani H., Dor J., Hourvitz A. (2011). Luteal phase oocyte retrieval and in vitro maturation is an optional procedure for urgent fertility preservation. Fertil. Steril..

[100] Chian R.C., Uzelac P.S., Nargund G. (2013). In vitro maturation of human immature oocytes for fertility preservation. Fertil. Steril..

[101] Blakemore J.K., Trawick E.C., Grifo J.A., Goldman K.N. (2020). Prognostic role of preimplantation genetic testing for aneuploidy in medically indicated fertility preservation. Fertil. Steril..

[102] Sciorio R., Anderson R.A. (2020). Fertility preservation and preimplantation genetic assessment for women with breast cancer. Cryobiology.

[103] Ghunaim S., Ghazeeri G., Khalife D., Azim H.A. (2020). Fertility preservation in patients with BRCA mutation. Ecancermedicalscience.

[104] Melnick A.P., Rosenwaks Z. (2018). Oocyte donation: Insights gleaned and future challenges. Fertil. Steril..

[105] Diaz-Garcia C., Domingo J., Garcia-Velasco J.A., Herraiz S., Mirabet V., Iniesta I., Cobo A., Remohí J., Pellicer A. (2018). Oocyte vitrification versus ovarian cortex transplantation in fertility preservation for adult women undergoing gonadotoxic treatments: A prospective cohort study. Fertil. Steril..

[106] Cobo A., García-Velasco J., Domingo J., Pellicer A., Remohí J. (2018). Elective and Onco-fertility preservation: Factors related to IVF outcomes. Hum. Reprod..

[107] Perachino M., Massarotti C., Razeti M.G., Parisi F., Arecco L., Damassi A., Fregatti P., Solinas C., Lambertini M. (2020). Gender-specific aspects related to type of fertility preservation strategies and access to fertility care. ESMO Open.

[108] Cobo A., García-Velasco J.A., Remohí J., Pellicer A. (2021). Oocyte vitrification for fertility preservation for both medical and nonmedical reasons. Fertil. Steril..

[109] Moravek M.B., Confino R., Lawson A.K., Smith K.N., Kazer R.R., Klock S.C., Gradishar W.J., Jeruss J.S., Pavone M.E. (2021). Predictors and outcomes in breast cancer patients who did or did not pursue fertility preservation. Breast Cancer Res. Treat..

[110] Oktay K., Turan V., Bedoschi G., Pacheco F.S., Moy F. (2015). Fertility Preservation Success Subsequent to Concurrent Aromatase Inhibitor Treatment and Ovarian Stimulation in Women With Breast Cancer. J. Clin. Oncol..

[111] Lawrenz B., Jauckus J., Kupka M., Strowitzki T., von Wolff M. (2010). Efficacy and safety of ovarian stimulation before chemotherapy in 205 cases. Fertil. Steril..

[112] Villarreal-Garza C., Mesa-Chavez F., de la Mora A.P., Miaja-Avila M., Garcia-Garcia M., Fonseca A., de la Rosa-Pacheco S., Cruz-Ramos M., Garza M.R.G., Mohar A. (2021). Prospective Study of Fertility Preservation in Young Women with Breast Cancer in Mexico. J. Natl. Compr. Cancer Netw..

[113] Herraiz S., Cervelló I. (2020). New insights for fertility preservation by ovarian tissue cryopreservation and transplantation in pediatric cancer patients. Fertil. Steril..

[114] Corkum K.S., Rhee D.S., Wafford Q.E., Demeestere I., Dasgupta R., Baertschiger R., Malek M.M., Aldrink J.H., Heaton T.E., Weil B.R. (2019). Fertility and hormone preservation and restoration for female children and adolescents receiving gonadotoxic cancer treatments: A systematic review. J. Pediatr. Surg..

[115] Wallace W.H., Kelsey T.W., Anderson R.A. (2016). Fertility preservation in pre-pubertal girls with cancer: The role of ovarian tissue cryopreservation. Fertil. Steril..

[116] (2014). Ovarian tissue cryopreservation: A committee opinion. Fertil. Steril..

[117] Oktay K., Harvey B.E., Loren A.W. (2018). Fertility Preservation in Patients With Cancer: ASCO Clinical Practice Guideline Update Summary. J. Oncol. Pract..

[118] Revel A., Revel-Vilk S., Aizenman E., Porat-Katz A., Safran A., Ben-Meir A., Weintraub M., Shapira M., Achache H., Laufer N. (2009). At what age can human oocytes be obtained?. Fertil. Steril..

[119] Feigin E., Abir R., Fisch B., Kravarusic D., Steinberg R., Nitke S., Avrahami G., Ben-Haroush A., Freud E. (2007). Laparoscopic ovarian tissue preservation in young patients at risk for ovarian failure as a result of chemotherapy/irradiation for primary malignancy. J. Pediatr. Surg..

[120] Michalczyk K., Cymbaluk-Płoska A. (2021). Fertility Preservation and Long-Term Monitoring of Gonadotoxicity in Girls, Adolescents and Young Adults Undergoing Cancer Treatment. Cancers.

[121] Donnez J., Dolmans M.M., Demylle D., Jadoul P., Pirard C., Squifflet J., Martinez-Madrid B., van Langendonckt A. (2004). Livebirth after orthotopic transplantation of cryopreserved ovarian tissue. Lancet.

[122] Gellert S.E., Pors S.E., Kristensen S.G., Bay-Bjørn A.M., Ernst E., Andersen C.Y. (2018). Transplantation of frozen-thawed ovarian tissue: An update on worldwide activity published in peer-reviewed papers and on the Danish cohort. J. Assist. Reprod. Genet..

[123] Donnez J., Dolmans M.M., Pellicer A., Diaz-Garcia C., Serrano M.S., Schmidt K.T., Ernst E., Luyckx V., Andersen C.Y. (2013). Restoration of ovarian activity and pregnancy after transplantation of cryopreserved ovarian tissue: A review of 60 cases of reimplantation. Fertil. Steril..

[124] Donnez J., Dolmans M.M., Diaz C., Pellicer A. (2015). Ovarian cortex transplantation: Time to move on from experimental studies to open clinical application. Fertil. Steril..

[125] Poirot C., Brugieres L., Yakouben K., Prades-Borio M., Marzouk F., de Lambert G., Pacquement H., Bernaudin F., Neven B., Paye-Jaouen A. (2019). Ovarian tissue cryopreservation for fertility preservation in 418 girls and adolescents up to 15 years of age facing highly gonadotoxic treatment. Twenty years of experience at a single center. Acta Obstet. Gynecol. Scand..

[126] Demeestere I., Simon P., Dedeken L., Moffa F., Tsépélidis S., Brachet C., Delbaere A., Devreker F., Ferster A. (2015). Live birth after autograft of ovarian tissue cryopreserved during childhood. Hum. Reprod..

[127] (2019). Fertility preservation in patients undergoing gonadotoxic therapy or gonadectomy: A committee opinion. Fertil. Steril..

[128] Martinez F. (2017). Update on fertility preservation from the Barcelona International Society for Fertility Preservation-ESHRE-ASRM 2015 expert meeting: Indications, results and future perspectives. Hum. Reprod..

[129] Sheshpari S., Shahnazi M., Mobarak H., Ahmadian S., Bedate A.M., Nariman-Saleh-Fam Z., Nouri M., Rahbarghazi R., Mahdipour M. (2019). Ovarian function and reproductive outcome after ovarian tissue transplantation: A systematic review. J. Transl. Med..

[130] Silber S. (2016). Ovarian tissue cryopreservation and transplantation: Scientific implications. J. Assist. Reprod. Genet..

[131] Herraiz S., Novella-Maestre E., Rodríguez B., Díaz C., Sánchez-Serrano M., Mirabet V., Pellicer A. (2014). Improving ovarian tissue cryopreservation for oncologic patients: Slow freezing versus vitrification, effect of different procedures and devices. Fertil. Steril..

[132] Shi Q., Xie Y., Wang Y., Li S. (2017). Vitrification versus slow freezing for human ovarian tissue cryopreservation: A systematic review and meta-anlaysis. Sci. Rep..

[133] Lee S., Ryu K.J., Kim B., Kang D., Kim Y.Y., Kim T. (2019). Comparison between Slow Freezing and Vitrification for Human Ovarian Tissue Cryopreservation and Xenotransplantation. Int. J. Mol. Sci.

[134] Fabbri R., Vicenti R., Macciocca M., Martino N.A., Dell’Aquila M.E., Pasquinelli G., Morselli-Labate A.M., Seracchioli R., Paradisi R. (2016). Morphological, ultrastructural and functional imaging of frozen/thawed and vitrified/warmed human ovarian tissue retrieved from oncological patients. Hum. Reprod..

[135] Wang T.R., Yan J., Lu C.L., Xia X., Yin T.L., Zhi X., Zhu X.H., Ding T., Hu W.H., Guo H.Y. (2016). Human single follicle growth in vitro from cryopreserved ovarian tissue after slow freezing or vitrification. Hum. Reprod..

[136] Dalman A., Farahani N.S.D.G., Totonchi M., Pirjani R., Ebrahimi B., Rezazadeh Valojerdi M. (2017). Slow freezing versus vitrification technique for human ovarian tissue cryopreservation: An evaluation of histological changes, WNT signaling pathway and apoptotic genes expression. Cryobiology.

[137] Hourvitz A., Yerushalmi G.M., Maman E., Raanani H., Elizur S., Brengauz M., Orvieto R., Dor J., Meirow D. (2015). Combination of ovarian tissue harvesting and immature oocyte collection for fertility preservation increases preservation yield. Reprod. Biomed. Online.

[138] Delattre S., Segers I., Van Moer E., Drakopoulos P., Mateizel I., Enghels L., Tournaye H., De Vos M. (2020). Combining fertility preservation procedures to spread the eggs across different baskets: A feasibility study. Hum. Reprod..

[139] Vuong L.N., Ho V.N.A., Ho T.M., Dang V.Q., Phung T.H., Giang N.H., Le A.H., Pham T.D., Wang R., Smitz J. (2020). In-vitro maturation of oocytes versus conventional IVF in women with infertility and a high antral follicle count: A randomized non-inferiority controlled trial. Hum. Reprod..

[140] Sato Y., Cheng Y., Kawamura K., Takae S., Hsueh A.J. (2012). C-type natriuretic peptide stimulates ovarian follicle development. Mol. Endocrinol..

[141] Kawamura K., Cheng Y., Kawamura N., Takae S., Okada A., Kawagoe Y., Mulders S., Terada Y., Hsueh A.J. (2011). Pre-ovulatory LH/hCG surge decreases C-type natriuretic peptide secretion by ovarian granulosa cells to promote meiotic resumption of pre-ovulatory oocytes. Hum. Reprod..

[142] Soto-Heras S., Menéndez-Blanco I., Catalá M.G., Izquierdo D., Thompson J.G., Paramio M.T. (2019). Biphasic in vitro maturation with C-type natriuretic peptide enhances the developmental competence of juvenile-goat oocytes. PLoS ONE.

[143] Zhang J., Wei Q., Cai J., Zhao X., Ma B. (2015). Effect of C-Type Natriuretic Peptide on Maturation and Developmental Competence of Goat Oocytes Matured In Vitro. PLoS ONE.

[144] Jia Z., Yang X., Liu K. (2021). Treatment of cattle oocytes with C-type natriuretic peptide before in vitro maturation enhances oocyte mitochondrial function. Anim. Reprod. Sci..

[145] Zhenwei J., Xianhua Z. (2019). Pre-IVM treatment with C-type natriuretic peptide in the presence of cysteamine enhances bovine oocytes antioxidant defense ability and developmental competence in vitro. Iran. J. Vet. Res..

[146] Zhao Y., Liao X., Krysta A.E., Bertoldo M.J., Richani D., Gilchrist R.B. (2020). Capacitation IVM improves cumulus function and oocyte quality in minimally stimulated mice. J. Assist. Reprod. Genet..

[147] Vuong L.N., Le A.H., Ho V.N.A., Pham T.D., Sanchez F., Romero S., De Vos M., Ho T.M., Gilchrist R.B., Smitz J. (2020). Live births after oocyte in vitro maturation with a prematuration step in women with polycystic ovary syndrome. J. Assist. Reprod. Genet..

[148] Plancha C.E., Rodrigues P., Marques M., Almeida J.M., Navarro-Costa P. (2021). The time is ripe for oocyte in vitro maturation. J. Assist. Reprod. Genet..

[149] Richani D., Wang X., Zeng H.T., Smitz J., Thompson J.G., Gilchrist R.B. (2014). Pre-maturation with cAMP modulators in conjunction with EGF-like peptides during in vitro maturation enhances mouse oocyte developmental competence. Mol. Reprod. Dev..

[150] Huang W., Nagano M., Kang S.S., Yanagawa Y., Takahashi Y. (2014). Prematurational culture with 3-isobutyl-1-methylxanthine synchronizes meiotic progression of the germinal vesicle stage and improves nuclear maturation and embryonic development in in vitro-grown bovine oocytes. J. Reprod. Dev..

[151] Van N.T.T., My L.B.A., Van Thuan N., Bui H.T. (2020). Improve the developmental competence of porcine oocytes from small antral follicles by pre-maturation culture method. Theriogenology.

[152] Fisch B., Abir R. (2018). Female fertility preservation: Past, present and future. Reproduction.

[153] Vilela J.M.V., Dolmans M.M., Amorim C.A. (2021). Ovarian tissue transportation: A systematic review. Reprod. Biomed. Online.

[154] Roness H., Meirow D. (2019). FERTILITY PRESERVATION: Follicle reserve loss in ovarian tissue transplantation. Reproduction.

[155] Gao J., Huang Y., Li M., Zhao H., Zhao Y., Li R., Yan J., Yu Y., Qiao J. (2015). Effect of Local Basic Fibroblast Growth Factor and Vascular Endothelial Growth Factor on Subcutaneously Allotransplanted Ovarian Tissue in Ovariectomized Mice. PLoS ONE.

[156] Kang B.J., Wang Y., Zhang L., Xiao Z., Li S.W. (2016). bFGF and VEGF improve the quality of vitrified-thawed human ovarian tissues after xenotransplantation to SCID mice. J. Assist. Reprod. Genet..

[157] Mahmoodi M., Mehranjani M.S., Shariatzadeh S.M., Eimani H., Shahverdi A. (2015). N-acetylcysteine improves function and follicular survival in mice ovarian grafts through inhibition of oxidative stress. Reprod. Biomed. Online.

[158] Manavella D.D., Cacciottola L., Pommé S., Desmet C.M., Jordan B.F., Donnez J., Amorim C.A., Dolmans M.M. (2018). Two-step transplantation with adipose tissue-derived stem cells increases follicle survival by enhancing vascularization in xenografted frozen-thawed human ovarian tissue. Hum. Reprod..

[159] Magen R., Shufaro Y., Daykan Y., Oron G., Tararashkina E., Levenberg S., Anuka E., Ben-Haroush A., Fisch B., Abir R. (2020). Use of Simvastatin, Fibrin Clots, and Their Combination to Improve Human Ovarian Tissue Grafting for Fertility Restoration after Anti-Cancer Therapy. Front. Oncol..

[160] Son W.Y., Henderson S., Cohen Y., Dahan M., Buckett W. (2019). Immature Oocyte for Fertility Preservation. Front. Endocrinol..

[161] Shapira M., Raanani H., Barshack I., Amariglio N., Derech-Haim S., Marciano M.N., Schiff E., Orvieto R., Meirow D. (2018). First delivery in a leukemia survivor after transplantation of cryopreserved ovarian tissue, evaluated for leukemia cells contamination. Fertil. Steril..

[162] Loren A.W., Mangu P.B., Beck L.N., Brennan L., Magdalinski A.J., Partridge A.H., Quinn G., Wallace W.H., Oktay K. (2013). Fertility preservation for patients with cancer: American Society of Clinical Oncology clinical practice guideline update. J. Clin. Oncol..

[163] Moghadam A.R.E., Moghadam M.T., Hemadi M., Saki G. (2021). Oocyte quality and aging. JBRA Assist. Reprod..

[164] Vo K.C.T., Kawamura K. (2021). In Vitro Activation Early Follicles: From the Basic Science to the Clinical Perspectives. Int. J. Mol. Sci..

[165] De Vos M., Devroey P., Fauser B.C. (2010). Primary ovarian insufficiency. Lancet.

[166] Kawamura K., Kawamura N., Hsueh A.J. (2016). Activation of dormant follicles: A new treatment for premature ovarian failure?. Curr. Opin. Obstet. Gynecol..

[167] Kawamura K., Cheng Y., Suzuki N., Deguchi M., Sato Y., Takae S., Ho C.H., Kawamura N., Tamura M., Hashimoto S. (2013). Hippo signaling disruption and Akt stimulation of ovarian follicles for infertility treatment. Proc. Natl. Acad. Sci. USA.

[168] Suzuki N., Yoshioka N., Takae S., Sugishita Y., Tamura M., Hashimoto S., Morimoto Y., Kawamura K. (2015). Successful fertility preservation following ovarian tissue vitrification in patients with primary ovarian insufficiency. Hum. Reprod..

[169] Hsueh A.J.W., Kawamura K. (2020). Hippo signaling disruption and ovarian follicle activation in infertile patients. Fertil. Steril..

[170] McLaughlin M., Albertini D.F., Wallace W.H.B., Anderson R.A., Telfer E.E. (2018). Metaphase II oocytes from human unilaminar follicles grown in a multi-step culture system. Mol. Hum. Reprod..

[171] O’Brien M.J., Pendola J.K., Eppig J.J. (2003). A revised protocol for in vitro development of mouse oocytes from primordial follicles dramatically improves their developmental competence. Biol. Reprod..

[172] Woodruff T.K. (2015). Oncofertility: A grand collaboration between reproductive medicine and oncology. Reproduction.

[173] Xiao S., Zhang J., Romero M.M., Smith K.N., Shea L.D., Woodruff T.K. (2015). In vitro follicle growth supports human oocyte meiotic maturation. Sci. Rep..

[174] Telfer E.E., Andersen C.Y. (2021). In vitro growth and maturation of primordial follicles and immature oocytes. Fertil. Steril..

[175] Tilly J.L., Telfer E.E. (2009). Purification of germline stem cells from adult mammalian ovaries: A step closer towards control of the female biological clock?. Mol. Hum. Reprod..

[176] White Y.A., Woods D.C., Takai Y., Ishihara O., Seki H., Tilly J.L. (2012). Oocyte formation by mitotically active germ cells purified from ovaries of reproductive-age women. Nat. Med..

[177] Hikabe O., Hamazaki N., Nagamatsu G., Obata Y., Hirao Y., Hamada N., Shimamoto S., Imamura T., Nakashima K., Saitou M. (2016). Reconstitution in vitro of the entire cycle of the mouse female germ line. Nature.

[178] Hayashi K., Ogushi S., Kurimoto K., Shimamoto S., Ohta H., Saitou M. (2012). Offspring from oocytes derived from in vitro primordial germ cell-like cells in mice. Science.

[179] Zhang H., Panula S., Petropoulos S., Edsgärd D., Busayavalasa K., Liu L., Li X., Risal S., Shen Y., Shao J. (2015). Adult human and mouse ovaries lack DDX4-expressing functional oogonial stem cells. Nat. Med..

[180] Horan C.J., Williams S.A. (2017). Oocyte stem cells: Fact or fantasy?. Reproduction.

[181] Luyckx V., Dolmans M.M., Vanacker J., Scalercio S.R., Donnez J., Amorim C.A. (2013). First step in developing a 3D biodegradable fibrin scaffold for an artificial ovary. J. Ovarian Res..

[182] Luyckx V., Dolmans M.M., Vanacker J., Legat C., Fortuño Moya C., Donnez J., Amorim C.A. (2014). A new step toward the artificial ovary: Survival and proliferation of isolated murine follicles after autologous transplantation in a fibrin scaffold. Fertil. Steril..

[183] Chiti M.C., Dolmans M.M., Mortiaux L., Zhuge F., Ouni E., Shahri P.A.K., Van Ruymbeke E., Champagne S.D., Donnez J., Amorim C.A. (2018). A novel fibrin-based artificial ovary prototype resembling human ovarian tissue in terms of architecture and rigidity. J. Assist. Reprod. Genet..

[184] Díaz-García C., Herraiz S. (2014). The artificial ovary: Any new step is a step forward. Fertil. Steril..

[185] Campo H., Cervelló I., Simón C. (2017). Bioengineering the Uterus: An Overview of Recent Advances and Future Perspectives in Reproductive Medicine. Ann. Biomed. Eng..

[186] Chiti M.C., Dolmans M.M., Orellana O., Soares M., Paulini F., Donnez J., Amorim C.A. (2016). Influence of follicle stage on artificial ovary outcome using fibrin as a matrix. Hum. Reprod..

[187] Pors S.E., Ramløse M., Nikiforov D., Lundsgaard K., Cheng J., Andersen C.Y., Kristensen S.G. (2019). Initial steps in reconstruction of the human ovary: Survival of pre-antral stage follicles in a decellularized human ovarian scaffold. Hum. Reprod..

[188] Laronda M.M., Rutz A.L., Xiao S., Whelan K.A., Duncan F.E., Roth E.W., Woodruff T.K., Shah R.N. (2017). A bioprosthetic ovary created using 3D printed microporous scaffolds restores ovarian function in sterilized mice. Nat. Commun..

[189] Laronda M.M. (2020). Engineering a bioprosthetic ovary for fertility and hormone restoration. Theriogenology.

[190] Dath C., Dethy A., Van Langendonckt A., Van Eyck A.S., Amorim C.A., Luyckx V., Donnez J., Dolmans M.M. (2011). Endothelial cells are essential for ovarian stromal tissue restructuring after xenotransplantation of isolated ovarian stromal cells. Hum. Reprod..

[191] Sittadjody S., Saul J.M., Joo S., Yoo J.J., Atala A., Opara E.C. (2013). Engineered multilayer ovarian tissue that secretes sex steroids and peptide hormones in response to gonadotropins. Biomaterials.

[192] Laronda M.M., Jakus A.E., Whelan K.A., Wertheim J.A., Shah R.N., Woodruff T.K. (2015). Initiation of puberty in mice following decellularized ovary transplant. Biomaterials.

[193] Woodard T.L., Bolcun-Filas E. (2016). Prolonging Reproductive Life after Cancer: The Need for Fertoprotective Therapies. Trends Cancer.

[194] Spears N., Lopes F., Stefansdottir A., Rossi V., De Felici M., Anderson R.A., Klinger F.G. (2019). Ovarian damage from chemotherapy and current approaches to its protection. Hum. Reprod. Update.

[195] Hao X., Anastácio A., Liu K., Rodriguez-Wallberg K.A. (2019). Ovarian Follicle Depletion Induced by Chemotherapy and the Investigational Stages of Potential Fertility-Protective Treatments-A Review. Int. J. Mol. Sci..

[196] Guzel Y., Bildik G., Oktem O. (2018). Sphingosine-1-phosphate protects human ovarian follicles from apoptosis in vitro. Eur. J. Obstet. Gynecol. Reprod. Biol..

[197] Meng Y., Xu Z., Wu F., Chen W., Xie S., Liu J., Huang X., Zhou Y. (2014). Sphingosine-1-phosphate suppresses cyclophosphamide induced follicle apoptosis in human fetal ovarian xenografts in nude mice. Fertil. Steril..

[198] González-Fernández B., Sánchez D.I., González-Gallego J., Tuñón M.J. (2017). Sphingosine 1-Phosphate Signaling as a Target in Hepatic Fibrosis Therapy. Front. Pharmacol..

[199] Wang E., He X., Zeng M. (2018). The Role of S1P and the Related Signaling Pathway in the Development of Tissue Fibrosis. Front. Pharmacol..

[200] Hancke K., Strauch O., Kissel C., Göbel H., Schäfer W., Denschlag D. (2007). Sphingosine 1-phosphate protects ovaries from chemotherapy-induced damage in vivo. Fertil. Steril..

[201] Zelinski M.B., Murphy M.K., Lawson M.S., Jurisicova A., Pau K.Y., Toscano N.P., Jacob D.S., Fanton J.K., Casper R.F., Dertinger S.D. (2011). In vivo delivery of FTY720 prevents radiation-induced ovarian failure and infertility in adult female nonhuman primates. Fertil. Steril..

[202] Kim S.Y., Cordeiro M.H., Serna V.A., Ebbert K., Butler L.M., Sinha S., Mills A.A., Woodruff T.K., Kurita T. (2013). Rescue of platinum-damaged oocytes from programmed cell death through inactivation of the p53 family signaling network. Cell Death Differ..

[203] Roti E.C.R., Salih S.M. (2012). Dexrazoxane ameliorates doxorubicin-induced injury in mouse ovarian cells. Biol. Reprod..

[204] Salih S.M., Ringelstetter A.K., Elsarrag M.Z., Abbott D.H., Roti E.C. (2015). Dexrazoxane abrogates acute doxorubicin toxicity in marmoset ovary. Biol. Reprod..

[205] Ganesan S., Keating A.F. (2016). The ovarian DNA damage repair response is induced prior to phosphoramide mustard-induced follicle depletion, and ataxia telangiectasia mutated inhibition prevents PM-induced follicle depletion. Toxicol. Appl. Pharmacol..

[206] Majidinia M., Sadeghpour A., Mehrzadi S., Reiter R.J., Khatami N., Yousefi B. (2017). Melatonin: A pleiotropic molecule that modulates DNA damage response and repair pathways. J. Pineal Res..

[207] Ting A.Y., Petroff B.K. (2010). Tamoxifen decreases ovarian follicular loss from experimental toxicant DMBA and chemotherapy agents cyclophosphamide and doxorubicin in the rat. J. Assist. Reprod. Genet..

[208] Tanaka Y., Kimura F., Zheng L., Kaku S., Takebayashi A., Kasahara K., Tsuji S., Murakami T. (2018). Protective effect of a mechanistic target of rapamycin inhibitor on an in vivo model ofcisplatin-induced ovarian gonadotoxicity. Exp. Anim..

[209] Madden J.A., Thomas P.Q., Keating A.F. (2017). Phosphoramide mustard induces autophagy markers and mTOR inhibition prevents follicle loss due to phosphoramide mustard exposure. Reprod. Toxicol..

[210] Jang H., Lee O.H., Lee Y., Yoon H., Chang E.M., Park M., Lee J.W., Hong K., Kim J.O., Kim N.K. (2016). Melatonin prevents cisplatin-induced primordial follicle loss via suppression of PTEN/AKT/FOXO3a pathway activation in the mouse ovary. J. Pineal Res..

[211] Hayun M., Naor Y., Weil M., Albeck M., Peled A., Don J., Haran-Ghera N., Sredni B. (2006). The immunomodulator AS101 induces growth arrest and apoptosis in multiple myeloma: Association with the Akt/survivin pathway. Biochem. Pharmacol..

[212] Kano M., Sosulski A.E., Zhang L., Saatcioglu H.D., Wang D., Nagykery N., Sabatini M.E., Gao G., Donahoe P.K., Pépin D. (2017). AMH/MIS as a contraceptive that protects the ovarian reserve during chemotherapy. Proc. Natl. Acad. Sci. USA.

[213] Mauri D., Gazouli I., Zarkavelis G., Papadaki A., Mavroeidis L., Gkoura S., Ntellas P., Amylidi A.L., Tsali L., Kampletsas E. (2020). Chemotherapy Associated Ovarian Failure. Front. Endocrinol..

[214] Findeklee S., Radosa J.C., Takacs Z., Hamza A., Sima R., Solomayer E., Sklavounos P. (2019). Fertility preservation in female cancer patients: Current knowledge and future perspectives. Minerva Ginecol..

[215] Lambertini M., Peccatori F.A., Demeestere I., Amant F., Wyns C., Stukenborg J.B., Paluch-Shimon S., Halaska M.J., Uzan C., Meissner J. (2020). Fertility preservation and post-treatment pregnancies in post-pubertal cancer patients: ESMO Clinical Practice Guidelines. Ann. Oncol..

[216] Dolmans M.M., Taylor H.S., Rodriguez-Wallberg K.A., Blumenfeld Z., Lambertini M., von Wolff M., Donnez J. (2020). Utility of gonadotropin-releasing hormone agonists for fertility preservation in women receiving chemotherapy: Pros and cons. Fertil. Steril..

[217] Oktay K., Harvey B.E., Partridge A.H., Quinn G.P., Reinecke J., Taylor H.S., Wallace W.H., Wang E.T., Loren A.W. (2018). Fertility Preservation in Patients With Cancer: ASCO Clinical Practice Guideline Update. J. Clin. Oncol..

[218] Deindl E., Zaruba M.M., Brunner S., Huber B., Mehl U., Assmann G., Hoefer I.E., Mueller-Hoecker J., Franz W.M. (2006). G-CSF administration after myocardial infarction in mice attenuates late ischemic cardiomyopathy by enhanced arteriogenesis. Faseb. J..

[219] Skaznik-Wikiel M.E., McGuire M.M., Sukhwani M., Donohue J., Chu T., Krivak T.C., Rajkovic A., Orwig K.E. (2013). Granulocyte colony-stimulating factor with or without stem cell factor extends time to premature ovarian insufficiency in female mice treated with alkylating chemotherapy. Fertil. Steril..

[220] Akdemir A., Zeybek B., Akman L., Ergenoglu A.M., Yeniel A.O., Erbas O., Yavasoglu A., Terek M.C., Taskiran D. (2014). Granulocyte-colony stimulating factor decreases the extent of ovarian damage caused by cisplatin in an experimental rat model. J. Gynecol. Oncol..

[221] Winarto H., Febia E., Purwoto G., Nuranna L. (2013). The need for laparoscopic ovarian transposition in young patients with cervical cancer undergoing radiotherapy. Int. J. Reprod. Med..

[222] Morgan R., Mimoun C., Lo Dico R. (2021). Ovarian transposition. J. Visc. Surg..

[223] Buonomo B., Multinu F., Casarin J., Betella I., Zanagnolo V., Aletti G., Peccatori F. (2021). Ovarian transposition in patients with cervical cancer prior to pelvic radiotherapy: A systematic review. Int. J. Gynecol. Cancer.

[224] Finch A., Valentini A., Greenblatt E., Lynch H.T., Ghadirian P., Armel S., Neuhausen S.L., Kim-Sing C., Tung N., Karlan B. (2013). Frequency of premature menopause in women who carry a BRCA1 or BRCA2 mutation. Fertil. Steril..

[225] Weinberg-Shukron A., Rachmiel M., Renbaum P., Gulsuner S., Walsh T., Lobel O., Dreifuss A., Ben-Moshe A., Zeligson S., Segel R. (2018). Essential Role of BRCA2 in Ovarian Development and Function. N. Engl. J. Med..

[226] Dou X., Guo T., Li G., Zhou L., Qin Y., Chen Z.J. (2016). Minichromosome maintenance complex component 8 mutations cause primary ovarian insufficiency. Fertil. Steril..

[227] Zhe J., Chen S., Chen X., Liu Y., Li Y., Zhou X., Zhang J. (2019). A novel heterozygous splice-altering mutation in HFM1 may be a cause of premature ovarian insufficiency. J. Ovarian Res..

[228] Weinberg-Shukron A., Renbaum P., Kalifa R., Zeligson S., Ben-Neriah Z., Dreifuss A., Abu-Rayyan A., Maatuk N., Fardian N., Rekler D. (2015). A mutation in the nucleoporin-107 gene causes XX gonadal dysgenesis. J. Clin. Investig..

[229] de Vries L., Behar D.M., Smirin-Yosef P., Lagovsky I., Tzur S., Basel-Vanagaite L. (2014). Exome sequencing reveals SYCE1 mutation associated with autosomal recessive primary ovarian insufficiency. J. Clin. Endocrinol. Metab..

[230] Islam N., Ugwoke S.P., Alhamdan R., Medrano J.H., Campbell B.K., Marsters P., Maalouf W.E. (2019). Steroids and miRNAs in assessment of ovarian tissue damage following cryopreservation. J. Mol. Endocrinol..

[231] Rodríguez-Iglesias B., Novella-Maestre E., Herraiz S., Díaz-García C., Pellicer N., Pellicer A. (2015). New methods to improve the safety assessment of cryopreserved ovarian tissue for fertility preservation in breast cancer patients. Fertil. Steril..

[232] Gudlevičienė Ž., Žilinskas K., Kundrotas G., Grubliauskaitė M., Baltriukienė D., Bukelskienė V. (2020). Slow-Freezing Cryopreservation Ensures High Ovarian Tissue Quality Followed by In Vivo and In Vitro Methods and Is Safe for Fertility Preservation. Medicina.

[233] Ayuandari S., Khasanah N., Riyanti I.W., Dewanto A., Sangun D.I.E., Wiweko B. (2021). Current Awareness and Attitude toward Fertility Preservation in Indonesia: A Nationwide Survey Among Health-care Providers. J. Hum. Reprod. Sci..

[234] Karim A.K.A., Ahmad M.F., Hamid H.A. (2021). Fertility preservation opportunities for cancer patients in Malaysia. Med. J. Malays..

[235] Akisada N., Monden N., Kishino T., Aoi J., Hayashi Y., Takahashi S., Nakamura M., Ishihara H., Nishizaki K. (2021). Otorhinolaryngologists/head and neck surgeons’ knowledge, attitudes, and practices regarding fertility preservation in young cancer patients treated with chemotherapy: An anonymous questionnaire survey. Int. J. Clin. Oncol..

[236] Hoffman A., Crocker L., Mathur A., Holman D., Weston J., Campbell S., Housten A., Bradford A., Agrawala S., Woodard T.L. (2021). Patients’ and Providers’ Needs and Preferences When Considering Fertility Preservation Before Cancer Treatment: Decision-Making Needs Assessment. JMIR Form. Res..

